# Lysyl hydroxylase 2 glucosylates collagen VI to drive lung cancer progression

**DOI:** 10.1172/JCI189197

**Published:** 2025-04-01

**Authors:** Shike Wang, Houfu Guo, Reo Fukushima, Masahiko Terajima, Min Liu, Guan-Yu Xiao, Lenka Koudelková, Chao Wu, Xin Liu, Jiang Yu, Emma Burris, Jun Xu, Alvise Schiavinato, William K. Russell, Mitsuo Yamauchi, Xiaochao Tan, Jonathan M. Kurie

**Affiliations:** 1 Department of Thoracic/Head and Neck Medical Oncology, The University of Texas-MD Anderson Cancer Center, Houston, Texas, USA.; 2 Department of Molecular and Cellular Biochemistry, University of Kentucky, Lexington, Kentucky, USA.; 3 Department of Biomedical Sciences, Adams School of Dentistry, University of North Carolina at Chapel Hill, Chapel Hill, North Carolina, USA.; 4 Department of Molecular and Cellular Oncology, Division of Basic Science Research, The University of Texas MD Anderson Cancer Center, Houston, Texas, USA.; 5 Department of Toxicology and Cancer Biology, The University of Kentucky, Lexington, Kentucky, USA.; 6 Cell and Molecular Biology, University of St Thomas, Houston, Texas, USA.; 7 Department of Molecular and Cellular Biology, The Advanced Cell Engineering and 3D Models Core, Baylor College of Medicine, Houston, Texas, USA.; 8 Center for Biochemistry, University of Cologne, Germany.; 9 Department of Biochemistry and Molecular Biology, The University of Texas Medical Branch, Galveston, Texas, USA.; 10 Department of Medicine, Tulane School of Medicine. New Orleans, Louisiana, USA.

**Keywords:** Cell biology, Oncology, Collagens, Integrins, Lung cancer

## Abstract

Lysyl hydroxylase 2 (LH2) is highly expressed in multiple tumor types and accelerates disease progression by hydroxylating lysine residues on fibrillar collagen telopeptides to generate stable collagen cross links in tumor stroma. Here, we show that a galactosylhydroxylysyl glucosyltransferase (GGT) domain on LH2 modified type-VI collagen (Col6) to promote lung adenocarcinoma (LUAD) growth and metastasis. In tumors generated by LUAD cells lacking LH2 GGT domain activity, stroma was less stiff, and stable types of collagen cross links were reduced. Mass spectrometric analysis of total and glycosylated peptides in parental and GGT-inactive tumor samples identified Col6 chain α3 (Col6a3), a component of the Col6 heterotrimeric molecule, as a candidate LH2 substrate. In gain- and loss-of-function studies, high Col6a3 levels increased tumor growth and metastatic activity and enhanced the proliferative, migratory, and invasive activities of LUAD cells. LH2 coimmunoprecipitated with Col6a3, and LH2 glucosylated Col6 in an in vitro reaction. Glucosylation increased the integrin-binding and promigratory activities of Col6 in LUAD cells. Col6a3 K2049 was deglucosylated in GGT-inactive tumor samples, and mutagenesis of Col6a3 K2049 phenocopied Col6a3 deficiency or LH2 GGT domain inactivation in LUAD cells. Thus, LH2 glucosylates Col6 to drive LUAD progression. These findings show that the GGT domain of LH2 is protumorigenic, identify Col6 as a candidate effector, and provide a rationale to develop pharmacological strategies that target LH2’s GGT domain in cancer cells.

## Abstract 2

LH2 is generally thought to exert pro-tumorigenic activity through its LH domain. These findings show that the GGT domain of LH2 is also pro-tumorigenic, identify Col6 as a candidate effector, and provide a rationale to develop pharmacological strategies that target LH2’s GGT domain in cancer cells.

## Graphical abstract

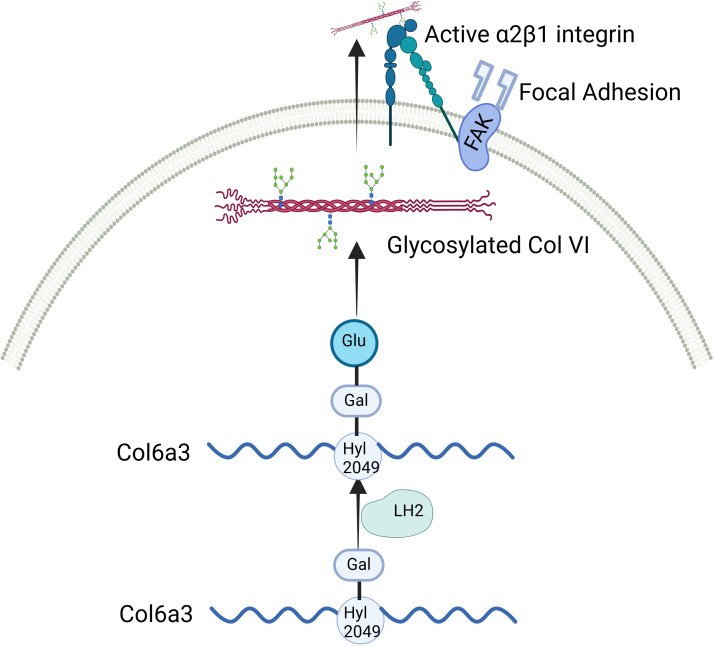

## Introduction

Advanced malignancies accumulate a dense collagenous stroma that is correlated with poor prognosis in multiple tumor types (1, 2). Underlying this association, fibrotic deposits within the tumor microenvironment (TME) are largely avascular, which creates a hypoxic milieu that inactivates antitumor immunity and initiates tumor-cell invasion (3). The collagenous matrix is composed of fibrillar and nonfibrillar collagens (4). Nonfibrillar collagens are often associated with the fibrillar (type I) collagen mesh. Collagen molecules function as ligands for receptors that initiate tumor cell invasion and facilitate immune escape, and they are stabilized by covalent intermolecular cross links that increase matrix stiffness and are associated with enhanced metastatic activity (5, 6). Thus, malignancies create a collagenous stroma that drives tumor progression through multifaceted processes.

In one working hypothesis, cancer cells are positioned at the apex of a prometastatic signaling hierarchy within the TME (7). In line with this concept, cancer cells express collagen modifying enzymes and proteases that remodel collagens deposited by intratumoral fibroblasts to create a metastasis permissive TME (8). During biosynthesis, collagens undergo a series of posttranslational modifications; specific lysine (Lys) residues are hydroxylated, and the resultant hydroxylysines (Hyl) can be mono or diglycosylated, resulting in galactosyl-hydroxylysine (G-Hyl) or glucosyl-galactosyl-hydroxylysine (GG-Hyl) (9). These modifications are catalyzed by lysyl hydroxylases (LH1-3), hydroxylysyl galactosyltransferases (GLT25D1/2), and the galactosylhydroxylysyl glucosyltransferase (GGT) LH3, respectively (10). Lys and Hyl residues in the nonhelical telopeptides can be further oxidatively deaminated by lysyl oxidases to initiate the collagen cross-linking process (11).These modifications impact the stability and biochemical properties of collagen (12), but the way in which they govern the biological properties of cancer cells is largely unknown.

Procollagen lysine 2-oxoglutarate 5-dioxygenase 2 (PLOD2) encodes LH2 and is a hypoxia-inducible gene that promotes metastasis in multiple tumor types (13). Contributing to its prometastatic activity, LH2 is unique among LH family members in its ability to hydroxylate Lys residues on collagen’s amino- and carboxy-terminal peptides (‘telopeptides’) and thereby generate stable Hyl-aldehyde–derived (Hyl^ald^-derived) collagen cross links (14). LH family members share a bifunctional domain structure consisting of LH and GGT domains, However, LH3 is generally considered the only LH family member to have a functional GGT domain (15). Challenging this traditional view, we detected GGT activity in LH1 and LH2, and structure-function studies showed that LH2’s GGT activity is restricted to an LH2 isoform (LH2b) that includes an alternatively spliced exon (exon 13A) (16). An inactivating mutation in the GGT domain abrogates the prometastatic activity of LH2b (16, 17).

While LH2 is generally thought to exert protumorigenic activity through its LH domain (18–20), we hypothesize that LH2 is a bifunctional enzyme and exerts prometastatic activity through both enzymatic domains. To test our hypothesis, here we sought to identify GGT domain substrates and determine how those substrates influence the collagenous stroma and impact metastasis in lung adenocarcinoma (LUAD) models.

## Results

### The GGT domain mediates the prometastatic activity of LH2.

**Figure 1:**
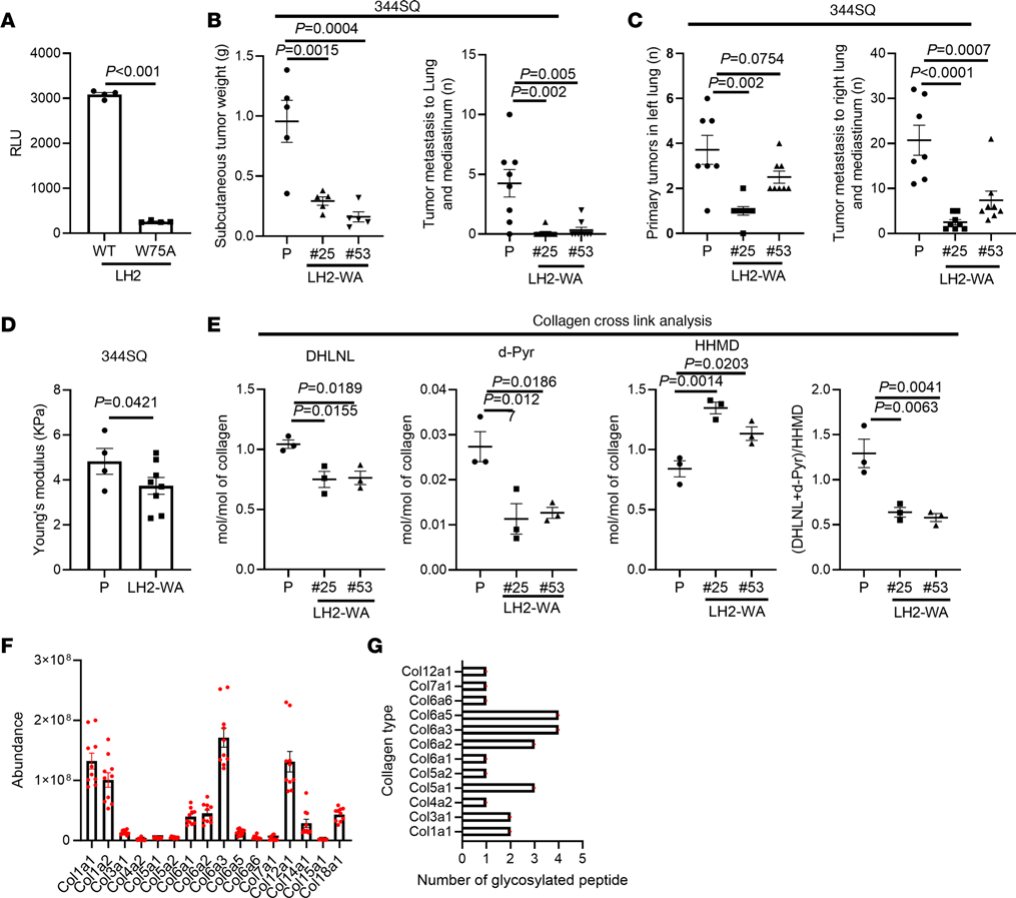
The GGT domain of LH2 drives LUAD progression and influences intratumoral collagen cross linking. (**A**) In vitro GGT activity assay. Purified WT or W75A-mutant LH2 protein samples (dots) reacted with galactosylhydroxylysine as substrate. (**B**) Flank tumor weights (left dot plot) and numbers of metastases to mediastinal nodes and contralateral lung (right dot plot) in syngeneic, immunocompetent mice (dots) injected subcutaneously with parental (P) or CRISPR/Cas-9–edited 344SQ cells bearing homozygous LH2 W75A mutations (LH2-WA). (**C**) Numbers of orthotopic lung tumors (left dot plot) and metastases to mediastinal nodes and contralateral lung (right dot plot). (**D**) Stiffness of flank tumors (dots) measured by atomic force microscopy. (**E**) Collagen cross links in collagen samples (dots) purified from flank tumor tissue. Dihydroxylysinonorleucine (DHLNL), deoxypyridinoline (d-Pyr), histidinohydroxymerodesmosine (HHMD). The ratio of stable (DHLNL + d-Pyr)–to–unstable (HHMD) cross-links. (**F**). Relative abundance of collagen family members in tumor tissues (dots). (**G**) Numbers of GG-Hyl residues identified per collagen family member in tumor samples from (**F**). *P* value was analyzed using Student’s 2-tailed *t* test (**A** and **D**) or 1-way ANOVA (**B**, **C**, and **E**).

To selectively disrupt GGT activity in LH2b, we carried out CRISPR/Cas-9–mediated mutagenesis of a conserved tryptophan (W75) that stabilizes UDP-glucose within the GGT domain’s active site (16). WT and mutant LH2b proteins were purified from 293T cells and reacted with UDP-glucose and a synthetic substrate, galactosylhydroxylysine (G-Hyl). Substrate glucosylation was measured based on free UDP release (16). Compared with WT LH2b, LH2 W75A was 10-fold less active (Figure 1A). This difference was not related to reduced stability of LH2 W75A (Supplemental Figure 1A; supplemental material available online with this article; https://doi.org/10.1172/JCI189197DS1). To assess the biological consequences of GGT inactivation, a LH2 W75A mutation (Supplemental Figure1, B and C) was introduced into 2 murine LUAD cell lines (344SQ, 344P) that are highly metastatic in syngeneic, immunocompetent mice (21). GGT inactivation had no detectable effect on LH2 protein levels (Supplemental Figure 1, D and E) but reduced the size and metastatic activity of primary tumors generated in the flank or lung (Figure 1, B and C and Supplemental Figure 1, F and G). Given that heightened metastatic activity results, in part, from intratumoral immunosuppression in this LUAD model (22), we quantified immune cell subsets in tumors generated by parental and GGT-inactive LUAD cells but detected no consistent differences (Supplemental Figure 2A). However, compared with parental tumors, GGT-inactive tumors were less stiff (Figure 1D) and demonstrated collagen cross-link alterations that result in reduced stiffness, including deficiencies in the Hyl^ald^-derived stable collagen cross links (e.g., dihydroxylysinonorleucine, deoxypyridinoline) and enrichment in the Lys^ald^-derived unstable collagen cross link histidinohydroxymerodesmosine (Figure 1E and Supplemental Figure 2B). Moreover, GGT-deficient LUAD cells exhibited impaired invasive properties in 3-dimensional matrices (Supplemental Figure 2, C and D). Thus, the GGT domain of LH2 modifies the collagenous matrix in ways that increase stromal stiffness and enhance the invasive and metastatic activities of LUAD cells.

### Col6 is a substrate of the LH2 GGT domain.

**Figure 2:**
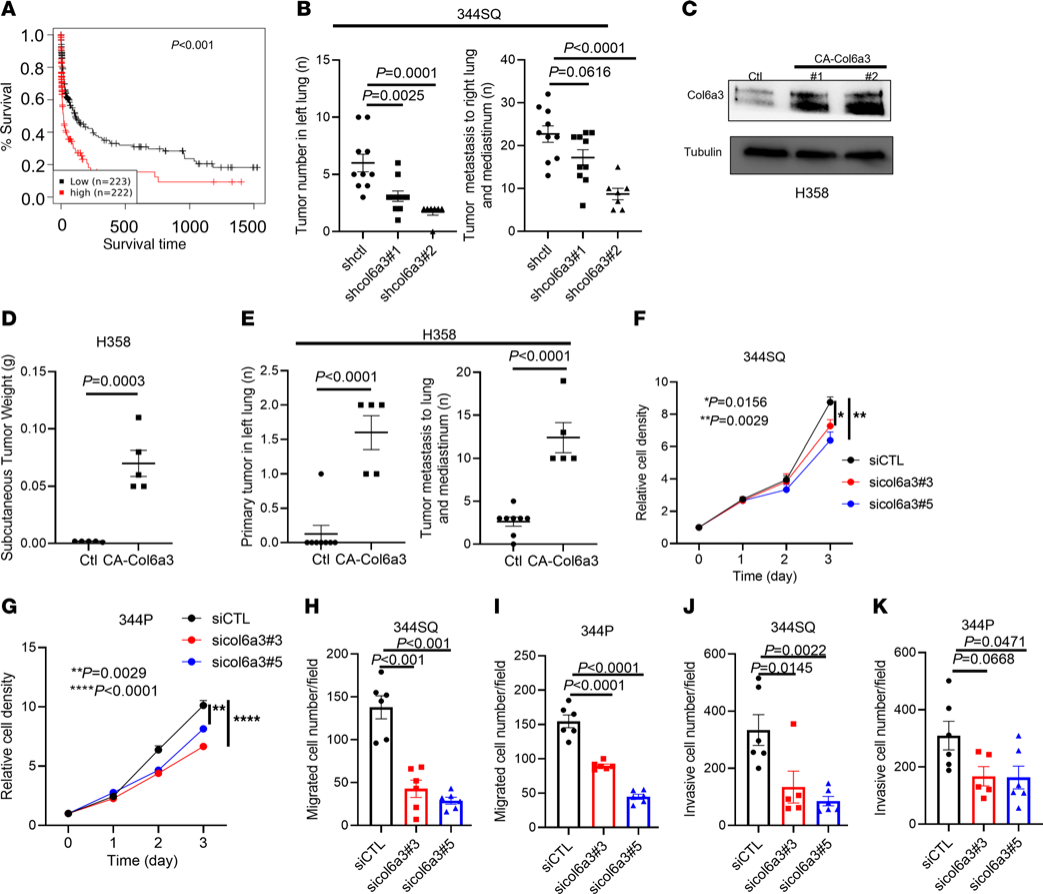
Heightened Col6a3 expression drives LUAD progression. (**A**) Kaplan-Meier survival analysis of patients with LUAD based on Col6a3 mRNA levels above (high) or below (low) the median value using GENT2 (http://gent2.appex.kr/gent2/) database. (**B**) Primary orthotopic lung tumors (left dot plot) and metastases to mediastinal nodes and contralateral lung (right dot plot) in syngeneic, immunocompetent mice injected with shRNA-transfected 344SQ cells. Control shRNA, shctrl. Col6a3 shRNA, shcol6a3. (**C**) Western blot (WB) confirmation that a CRISPR-mediated targeting approach activates endogenous Col6a3 expression in H358 cells. CRISPR-activated Col6a3 (CA-Col6a3) mass populations (numbers 1 and 2). Control transfectants (Ctl) were transfected with a vector in which guide RNAs were replaced with 20 noncoding nucleotides. (**D**) Flank tumor weights. Nu/nu mice injected with cells in **C**. (**E**) Numbers of orthotopic lung tumors (left dot plot) and metastases to mediastinal nodes and contralateral lung (right dot plot). Nu/nu mice injected with cells in **C**. (**F**–**K**) WST-1 proliferation assays (**F** and **G**) and Boyden chamber assays of migratory (**H** and **I**) and invasive (**J** and **K**) activities. For proliferation assays, mean values were calculated from replicate wells (dots). For Boyden chamber assays, mean values were calculated from multiple fields (dots) from replicate wells. *P* value was analyzed using Student’s 2-tailed *t* test (**D** and **E**) or 1-way ANOVA (**B** and **H**–**K**) or 2-way ANOVA (**F** and **G**).

To determine how the GGT domain of LH2 influences the collagenous stroma, we isolated total protein samples from flank tumors generated by parental and GGT-inactive LUAD cells and carried out liquid chromatography mass spectrometry (LC-MS). We identified a total of 4,379 proteins (Supplemental Table 1), 110 of which were present at different concentrations in parental and GGT-inactive tumors (Supplemental Figure 3, A and B). Based on Gene Ontology terms, the proteins downregulated in GGT-deficient tumors (*n* = 66) were enriched in terms related to extracellular matrix functions (Supplemental Figure 3C). Among the collagen family members identified (Figure 1F), LC-MS coverage varied from 14%–62%, and the total level of each collagen in parental and GGT-inactive tumors was not significantly different. Based on posttranslational modification analysis, Col6 family members were among the most highly glycosylated collagens (Figure 1G). Col6 is a triple helical molecule comprised of α1, α2, and α3 chains. In The Cancer Genome Atlas (TCGA) pancancer database, LUAD is one of multiple tumor types in which Col6a3 mRNA levels are higher in malignant than normal tissues (Supplemental Figure 4A), whereas Col6a1 and Col6a2 are not differentially expressed (Supplemental Figure 4B). Col6a3 mRNA levels are inversely correlated with survival durations in patients with LUAD (Figure 2A).

**Figure 3:**
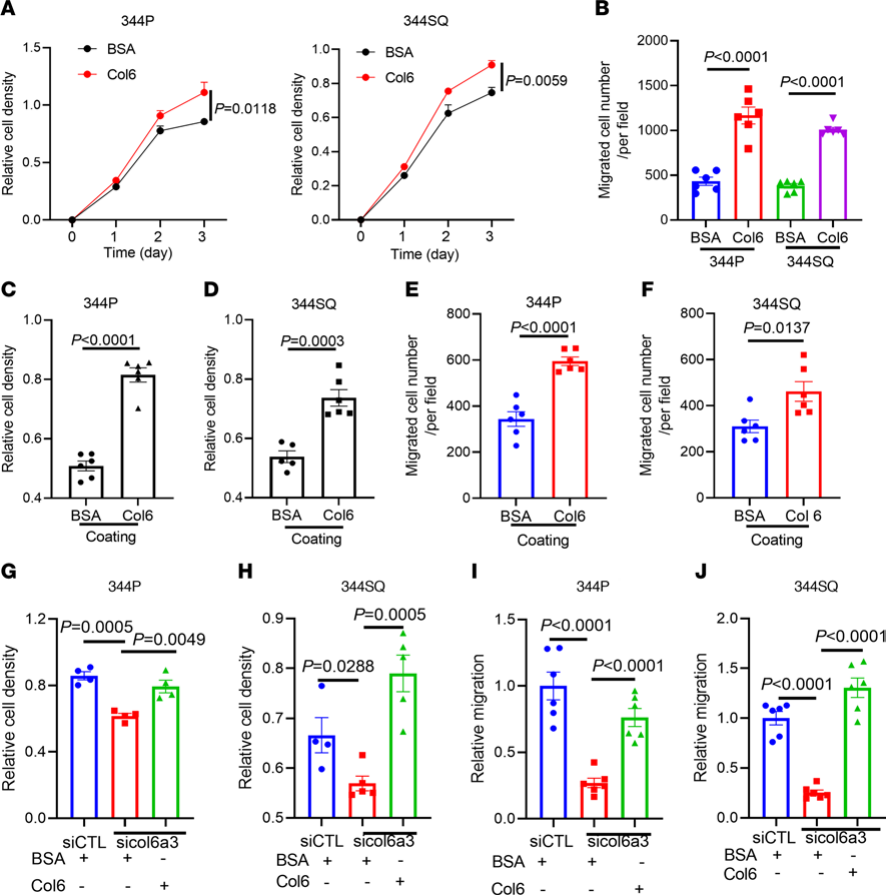
Col6 treatment enhances LUAD cell proliferation and migration. (**A**) WST-1 proliferation assays on 344P cells (left graph) and 344SQ cells (right graph) treated with soluble 10 μg/mL BSA or Col6. (**B**) Boyden chamber migration assays on 344SQ cells and 344P cells treated with soluble Col6 or BSA. (**C** and **D**) WST-1 proliferation assays on 344P cells (**C**) and 344SQ cells (**D**) seeded on Col6- or BSA-coated surfaces. (**E** and **F**) Boyden chamber migration assays on 344P cells (**E**) and 344SQ cells (**F**) seeded on Col6- or BSA-coated surfaces. (**G**–**J**) WST-1 proliferation assay (**G** and **H**) and Boyden chamber migration assay (**I** and **J**) on siRNA-transfected 344SQ cells treated with Col6 or BSA. For proliferation assays, mean values were calculated from replicate wells (dots). For Boyden chamber assays, mean values were calculated from multiple fields (dots) from replicate wells. *P* values were determined using Student’s 2-tailed *t* test (**B**–**F**) or 1-way ANOVA (**G**–**J**) or 2-way ANOVA (**A**).

To assess the biological role of Col6a3, we carried out short hairpin RNA–mediated (shRNA-mediated) Col6a3 depletion studies on 344SQ murine LUAD cells and CRISPR-mediated activation of endogenous Col6a3 expression (CA-Col6a3) in H358 human LUAD cells. In mice, tumors generated by Col6a3-deficient 344SQ cells were smaller and less metastatic than those generated by Col6a3-replete cells (Figure 2B and Supplemental Figure 4C), and tumors generated by CA-Col6a3 H358 cells were larger and more metastatic than tumors generated by control transfectants (Figure 2, C–E). To assess the biological role of Col6a3 in cultured cells, we carried out small interfering RNA–mediated (siRNA-mediated) depletion studies and found that Col6a3-deficient 344SQ cells exhibited reduced proliferative, migratory, invasive, and adhesive properties (Figure 2 F–K, and Supplemental Figure 5, A–F). Added in solution or as a substrate for cells in monolayer culture, purified Col6 protein exerted promigratory and proliferative activities (Figure 3, A–F, and Supplemental Figure 5, G and H). Soluble Col6 protein reversed the effect of siRNA-mediated Col6a3 depletion (Figure 3, G–J and Supplemental Figure 5I), confirming the proliferative and promigratory activities of Col6. Thus, Col6a3 exerts protumorigenic activities in LUAD.

**Figure 4:**
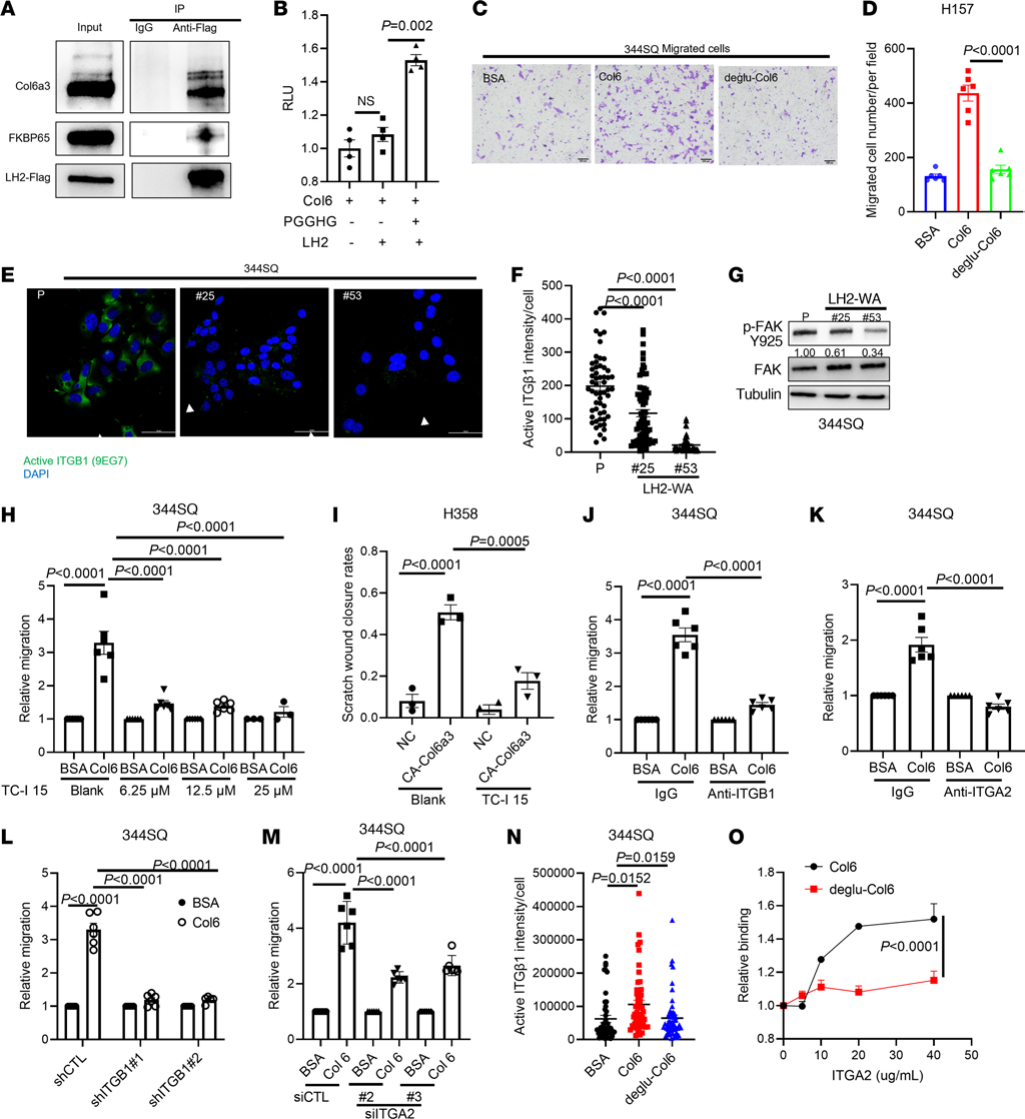
Glucosylation enhances the cell biological activities of Col6. (**A**) IP/WB analysis of H157 cells transfected with Flag-tagged LH2. Col6a3 coimmunoprecipitated with HA-tagged LH2. The LH2 chaperone FKBP65 included as a positive control. Total cell lysates (input). (**B**) In vitro GGT activity assay on purified LH2 reacted with PGGHG-pretreated Col6 as substrate. Each dot represents a replicate reaction. (**C** and **D**) Boyden chamber migration assays. 344P cells were seeded on membranes coated with Col6, PGGHG-pretreated Col6 (deglu-Col6), or BSA. Migrated cells were imaged (**C**) and quantified (**D**). (**E**) Immunocytochemical detection of activated ITGβ1 (arrows) in 344SQ cells. Scale bar 50 μm. (**F**) Quantification of activated ITGβ1 per cell (dot). (**G**) WB analysis of 344SQ cells. Densitometric quantification of p-FAK normalized based on total FAK (values under gel). (**H**) Boyden chamber migration assay on 344SQ cells treated with ITGα2 inhibitor TC-I 15 in the presence of soluble Col6 or BSA. (**I**) Scratch wound closure rates for control (NC) and CA-Col6a3 H358 cells in the presence or absence of TC-I 15. (**J** and **K**) Boyden chamber migration assays on 344SQ cells treated with neutralizing antibodies against ITGβ1 (**J**) or ITGα2 (**K**) followed by soluble Col6 or BSA. (**L** and **M**) Boyden chamber migration assays on shITGβ1- (**L**) or siITGα2- (**M**) transfected 344SQ cells treated with soluble Col6 or BSA. (**N**) Quantification of activated ITGβ1 per cell (dot). 344SQ cells were seeded on surfaces coated with BSA, Col6, or PGGHG-treated Col6 (deglu-Col6) and immunostained with anti-activated ITGβ1 antibody. (**O**) Solid phase binding assays on purified ITGα2/β1 heterodimers incubated with PGGHG-treated (deglu-Col6) or untreated Col6. *P* values were determined using 1-way ANOVA (**B**, **D**, **F**, and **H**–**N**) or 2-way ANOVA (**O**).

Col6 is highly glycosylated (23, 24), but the way in which the glycosylation state of Col6 is regulated and the effect of glycosylation on its biological properties remain unclear. The α1 and α2 chains are invariant members of the Col6 triple helix, but α3 can be replaced with α5 or α6 in α3-deficient cells (25). Total Col6α3 levels were not different in parental and GGT-inactive tumors, but GGT-inactive tumors had higher levels of Col6α5 and Col6α6 (Supplemental Figure 6, A–C), which led us to suspect that Col6α3 was functionally deficient in GGT-inactive tumors. To address functional interactions between LH2 and Col6a3, we carried out coimmunoprecipitation studies and found that Col6a3 immunoprecipitated from LUAD cells using LH2-Flag as bait (Figure 4A), and Col6a3 was identified as a LH2-interacting protein in the Pathway Commons Protein-Protein Interactions database (26). To test the hypothesis that Col6 is a LH2 GGT domain substrate, we reacted purified placental Col6 protein with LH2 and UDP-glucose, and substrate glucosylation was detected based on free UDP release (16). To increase the availability of substrate glucosylation sites, we pretreated Col6 with the collagen glucosidase protein-glucosylgalactosylhydroxylysine glucosidase (PGGHG), which enhances assay sensitivity (16). PGGHG deglucosylated sites on Col6a3 with variable efficiency (Supplemental Table 2). Under these conditions, LH2 increased UDP release, and activity was abolished by removal of PGGHG (Figure 4B), suggesting that the signal reflects LH2-dependent Col6 glucosylation. LH2 did not increase UDP release in reactions that lacked Col6 (Supplemental Figure 6D), arguing against PGGHG as a LH2 substrate.

### Glucosylation increases the protumorigenic properties of Col6.

To assess how glucosylation influences the biological activities of Col6, we treated LUAD cells with Col6 that had been pretreated with PGGHG and found that PGGHG pretreatment diminished the promigratory activity of Col6, whereas cell adhesion and proliferation were not affected (Figure 4, C and D and Supplemental Figure 7, A and B), suggesting that cell migration is uniquely dependent on the glucosylation state of Col6. Secreted Col6 binds to the collagen receptor NG2 and to specific integrin (ITG) heterodimeric complexes, including α1β1, α2β1, and α10β1, which are key focal adhesion (FA) components (27). In TCGA lung cancer cohorts, Col6a3 mRNA levels are correlated with each of these ITGs (Supplemental Figure 7C). The levels of activated, but not total, ITGβ1 and phosphorylated FA kinase were higher in parental than GGT-inactive LUAD cells (Figure 4, E–G and Supplemental Figure 7D). These findings were not due to reduced Col6 secretion (Supplemental Figure 7, E and F). Small molecules, neutralizing antibodies, and siRNAs against ITGα2/β1 attenuated LUAD cell migration driven by recombinant Col6 protein treatment or CRISPR-mediated activation of endogenous Col6a3 expression (Figure 4, H–M and Supplemental Figure 8), whereas depletion of NG2 or ITGα1 or α10 did not have this effect (Supplemental Figure 9, A–E). GGT inactivation did not detectably reduce ITGA2 levels (Supplemental Figure 9F). Instead, activated ITGβ1 levels were higher in LUAD cells seeded on glucosylated than deglucosylated Col6 (Figure 4N), and solid phase binding assays showed that glucosylation enhances the binding activity of Col6 to purified ITG α2/β1 heterodimers (Figure 4O and Supplemental Figure 9G). We conclude that glucosylation enhances the ITG-binding and promigratory activities of Col6.

**Figure 5:**
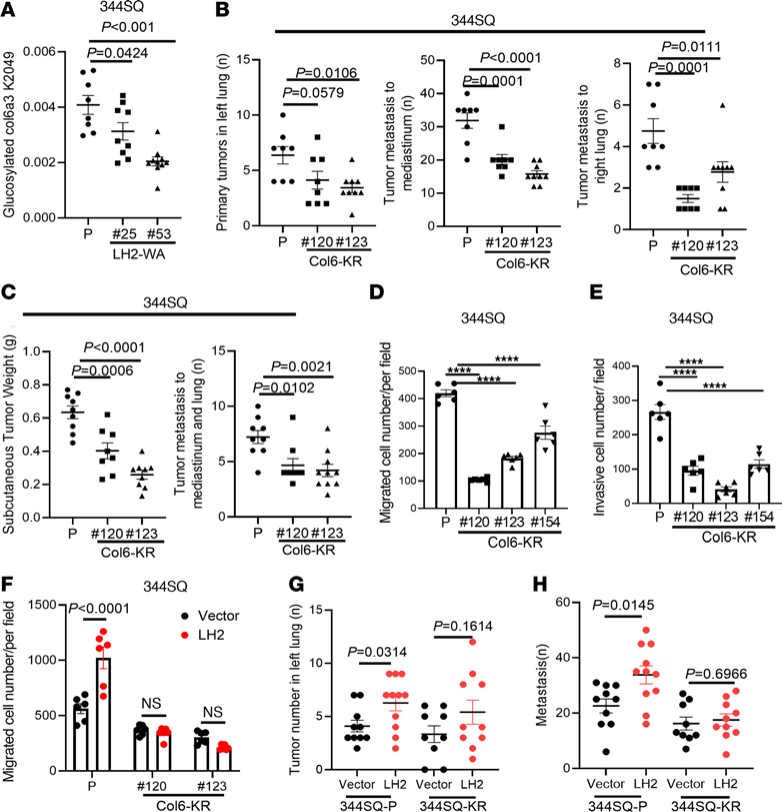
A Col6a3 K2049R mutation phenocopies LH2 GGT domain inactivation in LUAD. (**A**) Abundance of glucosylated Col6a3 K2049 peptides in parental (P) and LH2-WA tumor samples (dots). Values normalized to total Col6a3. (**B**) Numbers of orthotopic lung tumors (left dot plot) and metastases to mediastinal nodes (middle dot plot) and contralateral lung (right dot plot). Syngeneic, immunocompetent mice were injected intrathoracically with parental or CRISPR/Cas-9-edited 344SQ cells that bear homozygous Col6a3 K2049R mutations (Col6-KR). (**C**) Flank tumor weights (left dot plot) and numbers of metastases (right dot plot) in syngeneic, immunocompetent mice injected with parental or Col6-KR 344SQ cells. (**D** and **E**) Boyden chamber migration (**D**) and invasion (**E**) assays on parental and Col6-KR 344SQ cells. (**F**) Boyden chamber migration assays on parental or Col6-KR 344SQ cells that have ectopic expression of LH2 or empty vector (vector). (**G** and **H**) Numbers of orthotopic lung tumors (**G**) and metastases to mediastinal nodes and contralateral lung (**H**) in syngeneic, immunocompetent mice. *P* values were determined using 1-way ANOVA. ****P < 0.0001.

**Figure 6:**
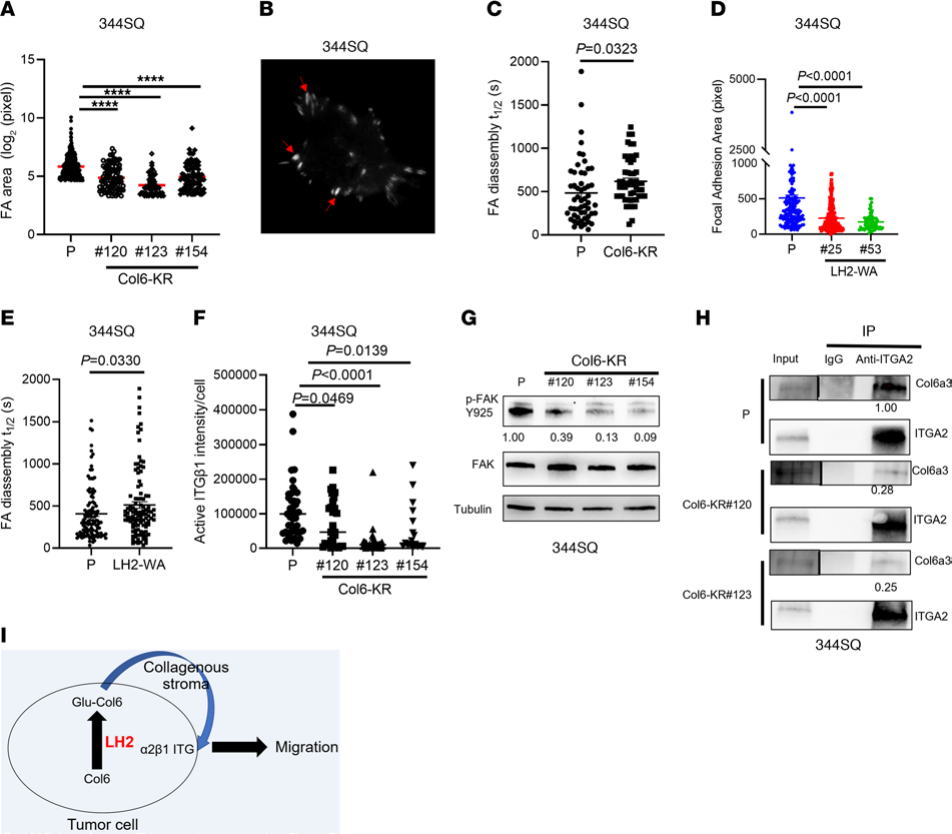
Col6a3 KR mutation inhibits FA dynamics. (**A**) Areas of FAs (dots) in anti-p-paxillin–stained parental (P) and Col6-KR 344SQ cells (n ≥ 50 per group). (**B**) Representative TIRF micrograph of mCherry-paxillin-transfected 344SQ cells to detect FAs (arrows), Original magnification, ×60. (**C**) Disassembly T_1/2_ of FAs (dots) in Col6-KR 344SQ cells determined based on 60 minute time-lapse sequences. (**D**) Areas of FAs (dots) in anti-p-paxillin–stained parental or LH2-WA 344SQ cells (n ≥ 50 per group). (**E**) Disassembly T_1/2_ of FAs (dots) in parental and LH2-WA 344SQ cells determined based on 60 minute time-lapse sequences. (**F**) Quantification of activated ITGβ1 per cell (dot) by immunocytochemical analysis with antiactivated ITGβ1 antibody. (**G**) WB analysis of phospho- and total FAK in parental and Col6-KR 344SQ cells. Densitometric values normalized based on total FAK are under gel. (**H**) IP/WB analysis of Col6a3/ITGα2. WB analysis to detect Col6a3 was carried out on anti-ITGα2–immunoprecipitated proteins isolated from parental or Col6-KR 344SQ cells. Relative densitometric values are under gels. Total cell lysates (input). (**I**) Schematic illustration of findings. *P* values were determined using 1-way ANOVA (**A**, **D**, and **F**) or Student’s 2-tailed *t* test (**C** and **E**). ****P < 0.0001.

To identify LH2 substrates on Col6, we quantified GG-Hyl on collagens in parental and GGT-inactive tumors. We detected GG-Hyl on multiple collagens, including Col1a1, Col3a1, Col4a2, Col5a1, Col5a2, Col6a1, Col6a3, and Col6a5. The glycosylated residues are listed in Supplemental Table 3. Of these collagens, only Col6a3 was differentially glycosylated in parental and GGT-inactive tumors (Figure 5A, Supplemental Figure 10, and Supplemental Figure 11A). Of the 5 reported Col6a3 glucosylation sites (28), we detected GG-Hyl at 3 sites (K2049, K2100, K2167), and GG-Hyl levels at 1 site (K2049) were significantly reduced in GGT-inactive tumors (Figure 5A and Supplemental Figure 10), whereas GG-Hyl levels at K2100 and K2167 were not different. Total Col6a3 levels in parental and GGT-inactive tumors were similar (Supplemental Figure 6A), arguing against Col6a3 loss as a contributor to the difference in GG-Hyl levels. To assess the biological role of Col6a3 K2049, we utilized CRISPR/Cas-9 mutagenesis to introduce a Col6a3 K2049R (Col6-KR) mutation in 344SQ cells (Supplemental Figure 11B). In vivo, Col6-KR cells generated orthotopic lung tumors and flank tumors that were smaller and less metastatic than those generated by parental cells (Figure 5, B and C). These differences were not due to reductions in Col6a3 protein levels (Supplemental Figure 11, C and D). Compared with parental cells, Col6-KR cells demonstrated reduced migratory and invasive activities (Figure 5, D and E and Supplemental Figure 11E). Ectopic LH2 expression accelerated cell migration and tumor progression to a greater extent in parental than Col6a3 K2049R cells (Figure 5, F–H and Supplemental Figure 11, F and G), indicating that Col6 K2049 is a critical effector of LH2. FAs were smaller and disassembled slower in Col6-KR than parental cells (Figure 6, A–C and Supplemental Video 1), a finding that was phenocopied in Col6a3-deficient and GGT-inactive LUAD cells (Figure 6, D and E, Supplemental Figure 12, A–C, and Supplemental Video 2). The levels of activated, but not total, ITGβ1 and phosphorylated FA kinase were higher in parental than Col6-KR LUAD cells (Figure 6, F and G and Supplemental Figure 12D). The KR mutation did not detectably reduce ITGA2 levels or Col6a3 secretion (Supplemental Figure 12, E and F). Instead, coimmunoprecipitation studies demonstrated that the ITGα2-binding activity of WT Col6 was higher than that of Col6-KR (Figure 6H). Thus, K2049 was critical for Col6 to bind ITG-α2, activate FA turnover, promote cell migration, and drive metastasis.

## Discussion

LH2 is generally thought to exert protumorigenic activity through its LH domain (18, 19, 29). Here, we show that the GGT domain of LH2 is also protumorigenic, and we identify Col6 as a candidate effector of the GGT domain. Col6 is highly glycosylated, but the way in which its glycosylation state is regulated and the impact of glycosylation on its biological properties remain unclear. Findings presented here suggest that Col6 is a LH2 substrate and that Col6 glucosylation facilitates ITG binding to increase LUAD cell migration (Figure 6I). By establishing Col6 as a LH2 substrate, we validate the GGT domain’s biochemical activity and the bifunctional nature of LH2. Furthermore, these findings provide biochemical insight into evidence that Col6 is a LH2 effector in sarcoma models (30).

A disaccharide (galactose-glucose) modification on Hyl located in the central helical domain is the primary glycosylation event on collagen (31). The extent to which glucosylation occurs varies widely among collagen family members (32). Type IV collagen in basement membranes is glycosylated to a greater extent than fibrillar (types I, II, III, V, XI, XXIV, and XXVII) collagens (33, 34). Glucosylation facilitates collagen secretion and fibril formation in the extracellular space (35), enhances receptor-binding activity of type I and type IV collagens (36–38), and influences collagen cross linking (39, 40). LH3 inactivation leads to sharp reductions in collagen glucosylation (41), which is the basis for the current belief that LH3 is the only mammalian collagen GGT. However, the disaccharide modification is conserved across collagens from sponges to humans, despite the absence of LH3 in the genomes of some invertebrates such as *C*. *elegans* (42). The findings reported here support the existence of collagen GGTs other than LH3. Given that GGT inactivation led to reduced Col6 glucosylation, we conclude that LH3 can not compensate for LH2 loss and that LH family members have nonoverlapping GGT activities. Nonoverlapping activities suggest that LH family members may have substrate specificities and function cooperatively in a unified GGT network.

Col6 plays a causal role in cancer progression. In multiple tumor types, Col6 levels are elevated, and high Col6 levels predict a worse clinical outcome (43). Col6 is a ligand for integrins that compose FAs (44), and ligand-binding initiates integrin endocytosis and FA dissociation (45). Compared with other Col6 family members, Col6a3 has unique carboxy-terminal sequences that undergo proteolytic cleavage, and the released fragments exert chemotactic forces on fibroblasts and macrophages within the TME to drive cancer progression (46). Although K2049 is not close to the proteolytic cleavage site on Col6a3, the findings presented here do not exclude the possibility that glucosylation influences proteolytic cleavage or the chemotactic properties of Col6a3.

Several shortcomings of our work warrant discussion. First, our LC/MS analysis of glycosylated proteins identified few differences between parental and GGT-inactive tumors. While this may reflect a specific role for LH2 as a regulator of Col6a3, a more likely explanation is that glycosylation events on fibrillar collagens, which generally contain fewer glycosylation sites than nonfibrillar collagens (e.g., type IV and VI), are below the detection limit of LC/MS. Detection could be improved by combining LC-MS/MS with other techniques, such as enrichment, chemical labeling, or enzymatic treatment. Second, our findings demonstrate cross-link alterations in GGT-inactive tumors, but we did not evaluate the mechanistic basis for this finding or the consequences of altered collagen cross linking on functional aspects of the TME that could have contributed to delayed tumor growth and metastasis suppression. Third, we did not investigate nonredundant roles of LH family members or how they function as components of a GGT network.

Our findings have potential clinical implications. Clinical trials carried out to reverse intratumoral fibrosis have failed to demonstrate clinical efficacy (47, 48). These trials, which targeted lysyl oxidases and specific matrix metalloproteinases, were based on an incomplete understanding of which patient populations are likely to benefit from such treatments (49). Findings presented here provide a rationale to develop selective antagonists of the LH2 GGT domain. Supporting the feasibility of such efforts, a high throughput approach to identify LH2 antagonists has been implemented utilizing a luciferase-based LH activity assay that we developed (50).

## Methods

### Sex as a biological variant.

Our study examined male mice because the syngeneic tumor model was derived using LUAD cells from male mice, and gender-mismatched tumor cells are rejected in this model.

### Cell lines and culture.

Murine and human LUAD cell lines (344SQ, 344P, and H358) were grown in a humidified atmosphere with 5% CO_2_ at 37°C in RPMI-1640 (Corning) supplemented with 10% FBS (Gibco). Human embryonic kidney 293T cells were grown in a humidified atmosphere with 5% CO_2_ at 37°C in DMEM supplemented with 10% FBS.

### Antibodies.

Antibodies against PLOD2 (Invitrogen, MA5-24145), ITG β1 (Genetex, GTX128839), ITG β1 (9EG7) (BD Pharmingen, 553715), ITG α2 (Invitrogen, MA5-35243), ITG α10 (Invitrogen, 2542S PA5-100840), NG2 (Proteintech, 55027-1-AP), paxillin (Cell Signaling Technology, 2542S), FAK Y925 (Cell Signaling Technology, 3284S), total FAK (Cell Signaling Technology, 3285S), tubulin (Sigma-Aldrich, T5168), FKBP65 (Cell Signaling Technology, 92445S), Col6a3 (Abcam ab231025), Flag (Sigma-Aldrich, F1804), his-tag (Proteintech 66005-1-Ig), murine HRP-conjugated secondary antibody (Cell Signaling Technology, 7076), rabbit HRP-conjugated secondary antibody (Cell Signaling Technology, 7074), Alexa Fluor-tagged secondary antibodies (Invitrogen, A-11008, A-11011), neutralizing ITG β1 (Thermo Fisher Scientific, 16-0291-85), neutralizing ITG α2 (Thermo Fisher Scientific 14-0491-82), murine IgG (Santa Cruz, sc-2025), rabbit IgG (Cell Signaling Technology, 2729), and Armenian Hamster IgG Isotype Control (Thermo Fisher Scientific 14-4888-81) were used.

### shRNAs, siRNAs, and plasmids.

The shRNAs and siRNAs were purchased from Sigma-Aldrich. Murine shITGB1 (TRCN0000066647 and TRCN0000178607), murine siItga1 (SASI_Mm01_00102775 and SASI_Mm02_00288356), mouse siNG2 (SASI_Mm01_00180514, SASI_Mm01_00180516, SASI_Mm01_00180517, SASI_Mm01_00180518 and SASI_Mm01_00180520), murine siITGA2 (SASI_Mm02_00313827, SASI_Mm01_00097732, SASI_Mm01_00097733, SASI_Mm01_00097734 and SASI_Mm02_00313828), murine siITGA10 (SASI_Mm02_00294711, SASI_Mm02_00294712, SASI_Mm02_00294713, SASI_Mm02_00294714 and SASI_Mm02_00294715) were used. The human COL6A3 CRISPR activation plasmid (sc-402899-ACT) and Control CRISPR Activation Plasmid (sc-437275) were purchased from Santa Cruz.

### Gene editing.

The synthesized guide RNAs (listed in Supplemental Table 4) were assembled into RNP complex with recombinant Cas9 proteins and electroporated into cells with the donor ssODN (listed in Supplemental Table 4). Electroporated cells were allowed to recover for 48 hours before being limiting dilution-plated at less-than 1 cell per well into 96-well plates in complete medium (RPMI +10%FBS). Once clones grew to sufficient sizes, they were expanded into 24-well plates for genomic DNA extraction and PCR amplification. The PCR products were digested with HhaI or PmlI to identify possible knock-in clones. Knock-in clones were verified by Sanger sequencing.

### CRISPR-mediated activation of endogenous Col6a3 expression.

To upregulate endogenous Col6a3 gene expression, we used a synergistic activation mediator transcription activation system. 293T cells were cotransfected with lentiviral packaging plasmids (pMD2.G and psPAX2) and a human COL6A3 CRISPR activation plasmid. A control vector lacking guide RNA contained 20 nucleotides of noncoding scrambled RNA sequence. Conditioned medium samples containing lentivirus particles were collected and utilized to infect H358 cells. After 48 hours, puromycin (2 μg/mL) was added. After 14 days in selection, mass populations were isolated.

### Animal experiments.

WT 129sv mice and nu/nu mice at least 8 weeks old were used for the experiments. Subcutaneous injections of 1 × 10^6^ cells in single-cell suspension were placed in the posterior flank in a volume of 100 μL of PBS. Orthotopic lung tumors were generated by intrathoracic injection of 1 × 10^6^ human lung cancer cells in single-cell suspension in a volume of 50 μL of PBS. Animals were monitored regularly and euthanized when they exhibited signs of morbidity or when the size of the subcutaneous tumor reached 1 cm in diameter, which occurred typically at 4–6 weeks’ time.

### Collagen cross-link analysis.

Collagen cross-link analysis was performed as reported (10). Briefly, collagen was reduced by standardized NaB^3^H_4_. Reduced collagen was hydrolyzed with 6 N HCl, then subjected to amino acid and cross-link analysis as described previously (11). The levels of the major immature reducible, dihydroxylysinonorleucine (DHLNL), hydroxylysinonorleucine (HLNL), and histidinehydroxymerodesmosine (HHMD), and mature nonreducible cross-links, pyridinoline (Pyr), deoxypyridinoline (d-Pyr) were quantified as moles/mole of collagen.

### LC-MS analysis.

Lyophilized tumor samples were suspended in 5% SDS, 50 mM TEAB (pH 7.55). The sample was then centrifuged at 17,000*g* for 10 minutes to remove any debris. Proteins were reduced by making the solution 20 mM TCEP (Thermo Fisher Scientific, 77720) and incubated at 65°C for 30 minutes. The sample was cooled to room temperature and 1 μL of 0.5 M iodoacetamide acid added and allowed to react for 20 minutes in the dark. 2.75 μL of 12% phosphoric acid was added to the protein solution. 165 μL of binding buffer (90% Methanol, 100 mM TEAB final; pH 7.1) was then added to the solution. The resulting solution was added to S-Trap spin column (protifi.com) and passed through the column using a bench top centrifuge (30 second spin at 4,000*g*). The spin column was washed with 400 μL of binding buffer and centrifuged. This was repeated 2 more times. Trypsin was added to the protein mixture in a ratio of 1:25 in 50 mM TEAB, pH 8, and incubated at 37°C for 4 hours. Peptides were eluted with 80 μL of 50 mM TEAB, followed by 80 μL of 0.2% formic acid, and finally 80 μL of 50% acetonitrile, 0.2% formic acid. The combined peptide solution was then dried in a speed vac and resuspended in 2% acetonitrile, 0.1% formic acid, 97.9% water and placed in an autosampler vial.

Digested proteins were analyzed by nanoLC-MS/MS (nanoRSLC, Thermo Fisher Scientific) using an Aurora series (Ion Opticks) reversed phase HPLC column (25 cm length × 75 μm inner diameter) directly injected to an Orbitrap Eclipse using a 120 min gradient (mobile phase A = 0.1% formic acid (Thermo Fisher Scientific Scientific), mobile phase B = 99.9% acetonitrile with 0.1% formic acid (Thermo Fisher Scientific); hold 12% B for 5 minutes, 2%–6% B in 0.1 minute, 6%–25% in 100 minutes, 25%–50% in 15 minutes) at a flow rate of 350 nL/min. Eluted peptide ions were analyzed using a data-dependent acquisition (DDA) with resolution settings of 120,000 and 15,000 (at m/z 200) for MS1 and MS2 scans, respectively. Tandem mass spectra utilized stepped collision energy HCD fragmentation (25%/35%/45%) normalized collision energy and were analyzed according to a label-free proteomic strategy using Proteome Discoverer (version 2.5.0.400, Thermo Fisher Scientific) with the Byonic (version 4.5-53 Protein Metrics), and searched against the mouse FASTA proteome. Mass tolerances of 10 ppm and 20 ppm were used for matching parent and fragment masses, respectively. Mass spectra were searched with a fixed modification of carbamidomethyl (C), and variable modifications of oxidation (M, K, and P), Galactosyl +178.048 Da (K), and Glucosylgalactosyl +340.101 Da (K).). Peptide spectral matches (PSMs) were filtered for high quality identifications (PEP2D < 0.01, Byonic score > 100, Delta Mod score > 10).

### GGT activity assay.

GGT activity was measured in reaction buffer (100 mM HEPES buffer pH 8.0, 150 mM NaCl) at 37°C for 1 hour with 1 μM LH_2_, 20 μM MnCl_2_,100μM UDP-glucose (Sigma-Aldrich), 1 mM dithiothreitol, 0.02% BSA, and 1 mM galactosyl hydroxylysine (Cayman Chemical) or 2 μg PGGHG-treated Col6. GGT activity was measured by detecting UDP production with an ATP-based luciferase assay (Promega) according to manufacturers’ manual.

### Cell proliferation assay.

Cells were seeded in 96-well plates (2 × 10^3^ cells/well) and incubated for defined time points. Cells were treated with 10 μg/mL BSA or soluble Col6 or seeded on surfaces coated with 100 μg/mL BSA or Col6. Relative cell densities of 4 replicates per condition were measured using the WST-1 reagent (Roche).

### 3-dimensional cell culture assay.

2 × 10^4^ cells were suspended singly in complete medium and then mixed with 1 mg/mL collagen I and 5% Matrigel. The mixture was plated into 24-well plates. After 48 hours, sphere morphology was imaged. The lengths of invasive projections were measured by Image J.

### Cell migration and invasion assays.

Cells (1 × 10^5^) in FBS-free RPMI-1640 were seeded in the upper wells (in triplicate) of Transwell and Matrigel chambers (Falcon) and allowed to migrate or invade, respectively, toward RPMI-1640 containing 10% FBS in the bottom wells. Where indicated, cells were seeded on membranes coated with 100 μg/mL BSA or Col6 or treated with 10 μg/mL BSA or Col6 or 50 μg/mL neutralizing antibodies or IgG. After 18 hours, migrating or invading cells were fixed and stained with 0.1% crystal violet (Sigma-Aldrich), photographed, and counted manually. Mean values were calculated from multiple fields from replicate wells.

### Wound healing assay.

Cells were seeded into 6-well plates (1 × 10^6^ cells/well, triplicates per group) and allowed to grow to confluence. A scratch was made using a 10 μL pipet tip. Floating cells were washed away by 1 × PBS. Cells were imaged immediately (0 hours) and after 24 hours in complete medium with or without 10 μM TC-I 15 (24 hours). Scratch widths at 0 hours and 24 hours were measured by Image J. The scratch wound closure rates were calculated (W_(0h)_–W_(24h)_)/ W_(0h)_. Mean values were calculated from replicate wells.

### Cell adhesion assay.

1 × 10^5^ cells were seeded on 96-well plates coated with or without type I collagen (100 μg/mL) and incubated for 1 to 2 hours. After the attached cells were washed twice with PBS, they were stained with 0.1% crystal violet and optical density of 4 replicates per condition was measured at 595 nm.

### Western blotting.

Cells were washed with PBS and then lysed to extract total proteins with cell lysis RIPA buffer (Cell Signaling Technology). Cell lysates were separated by SDS-PAGE, transferred onto Nitrocellulose Transfer Membrane (Whatman Schleicher & Schuell), and then incubated with primary antibodies and HRP-conjugated secondary antibodies (Cell Signaling Technology). Protein bands were visualized with Pierce ECL Western Blotting substrate (Thermo Fisher Scientific).

### Quantitative PCR.

Total RNA was purified from cells using RNeasy Plus Kits (QIAGEN) according to the manufacturer’s protocol. The mRNA levels were quantified using a SYBRGreen-based system (Applied Biosystems) after reverse transcription with qScript cDNA SuperMix (Quanta). mRNA levels were normalized based on ribosomal protein L32 (Rpl32) mRNA levels. See Supplemental Table 5 for primer sequences.

### Immunofluorescent staining.

Cancer cells were cultured on glass coverslips, fixed with formaldehyde, permeabilized with 0.5% Triton X-100 in PBS, and incubated with primary antibody followed by Alexa Fluor-conjugated secondary antibody. Cells were analyzed using an A1+ platform (Nikon Instruments) confocal microscopy equipped with 63×/1.4 NA Oil, 100×/1.45 NA Oil, and 20×/0.75 NA Air objectives, 405/488/561 nm laser lines, GaAsP detectors, and Okolab stage top incubator. Images were acquired using NIS- Elements software (Nikon instruments).

### Total internal reflection fluorescence imaging.

Cells were plated on type I collagen-coated glass bottom 35 mm plates (Corning). Cells were transfected with mCherry-paxillin. After 2 days in culture, medium was replaced with live cell imaging solution (Invitrogen). Time-lapse TIRF was performed on the OMX Blaze V4 SIM Super- Resolution microscope equipped with a TIRF objective (60 ×).

### Image processing and quantitative analysis.

For fixed cell imaging, the raw images were analyzed in Fiji/ImageJ (https://imagej.nih.gov/ij/download.html). For live cell imaging, the raw images were processed/analyzed in Imaris 9.6 (Bitplane software, Oxford instruments) with MATLAB XTensions and Fiji/ImageJ. Time-lapse videos were analyzed for FA disassembly assays in Imaris spots tracking module in Surpass mode. Disassembly rate constants and half-life values were calculated according to a previous study (51).

### Tumor IHC analysis.

Four-μm tissue sections from formalin-fixed and paraffin-embedded lung tumor tissues were stained using an automated immunostainer platform, the Leica Bond Max automated stainer (Leica Biosystems Nussloch GmbH). Following the Leica Bond protocol, the tissue sections were deparaffinized and rehydrated. Antigen retrieval was performed using Bond Solution #2 (Leica Biosystems, equivalent EDTA, pH 9.0) for 30 minutes. Primary antibodies (Col6a3, 20 μg/mL, Abcam) were incubated at room temperature. The primary antibody was detected using the Bond Polymer Refine Detection kit (Leica Biosystems) with DAB as the chromogen. Slides were counterstained with hematoxylin, dehydrated, and cover slipped. Immunostained sections were digitally scanned using the Aperio AT2 slide scanner (Leica Biosystems) under 20 × objective magnification. Digital image analysis was performed using ImageScope software containing a pathologist-trained specific algorithm (Positive Pixel Count V9).

### Tumor stiffness assay.

Atomic force microscopy (AFM) measurements were conducted in the AFM Core Facility at the University of Texas Health Science Center at Houston using a BioScope IITM Controller (Bruker Corporation). OCT-embedded tumors were cut to 25 μm frozen sections and deposited on poly L-lysine-coated glass slides and kept frozen to –20°C. Tissue sections were thawed at room temperature for 20 minutes. OCT was then rinsed with PBS for 20 minutes and slides were immediately frozen until AFM experiments. Tissue samples were rehydrated for at least 15 minutes prior to AFM measurements. Data were collected in PBS using a Bruker BioScope II Controller (Bruker Corporation) integrated with a Nikon TE2000-E inverted optical microscope (Nikon Instruments). Force curves from at least 20 randomly selected points per tumor section were taken using Novascan colloidal AFM probes. These probes consisted of a 5-μm-diameter borosilicate glass particle attached to the edge of a silicon nitride V-shaped cantilever, with a nominal spring constant of 0.24 N/m. The cantilever was calibrated for its laser sensitivity using the thermal oscillation method prior to each experiment. Indentation curves were captured using 4-μm ramp sizes, a scan rate of 0.5 Hz, and a trigger threshold with a maximum load of 10 nN. The Young’s modulus was calculated following the Hertz model (spherical indenter radius = 2.5 μm) with a Poisson’s ratio of 0.5, using the NanoScope Analysis software version 1.5 (*Protein expression and purification*. PGGHG was expressed in *E*. *coli* strain Rosetta (DE3). Cells expressing PGGHG were induced with 1 mM isopropyl β-D-1-thiogalactopyranoside (IPTG) for 16 hours at 16 °C. Cells were collected, pelleted, and resuspended in binding buffer (20 mM Tris, pH 8.0, 200 mM NaCl and 15 mM imidazole). The cells were lysed by sonication and centrifuged at 23,000*g* for 15 minutes. The recombinant PGGHG were purified with immobilized metal affinity chromatography. Human WT and mutant LH2 were expressed in 293T cells with plasmids pLVX-wt LH2-Flag and pLVX-w75a LH2-Flag and pulled down by Anti-FLAG M2 Affinity Gel (Sigma-Aldrich) and eluted by 3 × flag peptide (100 μg/mL) (Sigma-Aldrich). His-tagged ectodomain ITG α2 was expressed in 293T cells utilizing the plasmid ITGA2-bio-His (Addgene, 51910) then were purified with his-tagged protein purification gel (MBL).

### Solid phase binding assay.

96-well plates were coated with Col6 (100 μg/mL). Col6 coating was deglucosylated with recombinant 100 μg PGGHG. Purified ITG-α2/β1 heterodimeric complexes were incubated with Col6 at room temperature for 2 hours. The bound ITG α2 was measured with HRP-conjugated anti-His antibody and detected with color development reagent (Abcam). Absorbance was read at 450 nm with a plate reader (Biotek) to quantify binding capacity.

### Immunoprecipitation assay.

Cells were lysed in cell lysis RIPA buffer. Supernatants were incubated with primary antibody and protein G agarose beads (Cell signaling Technology) at 4°C overnight. The immune complex was washed with 1 × RIPA buffer 4 times and boiled in 1 × SDS loading buffer at 98°C for 10 minutes. The resulting samples were subjected to Western blot analysis.

### Statistics.

Results shown are representative of replicated experiments and are the mean ± SEM from at least triplicate samples or randomly chosen cells within a microscopic field. Statistical evaluations were carried out with Prism 9 (GraphPad Software, Inc.). *P* values were analyzed using Unpaired 2-tailed Student’s *t* tests or ANOVA for 2 or more groups, respectively, and *P* < 0.05 were considered statistically significant. Kaplan-Meier survival analysis were performed in GENT2 database (52).

### Study approval.

All animal experiments were reviewed and approved by the Institutional Animal Care and Use Committee at The University of Texas MD Anderson Cancer Center.

### Data availability.

All data associated with this study are present in the manuscript or in the supplemental materials and are available in the Supporting Data Values file.

## Author contributions

JMK and SW wrote the paper. SW conceived, designed, executed, and interpreted the molecular biology, cell biology, and in vivo experiments. JMK and XT conceived and supervised the project and contributed to the design and interpretation of all experiments. HG and ML executed the protein purification. RF, MT, and MY conceived, designed, executed and interpreted the collagen cross-link analysis. GX and LK assisted SW with the TIRF experiment. JX and CW conceived, designed, and executed gene editing. XL assisted SW with the in vivo experiments. JY bred the mice for the in vivo studies. EB assisted SW with the adhesion experiments. WKR directed and interpreted the LC-MS experiments. AS assisted with Col6a3 biochemical analysis.

## Supplementary Material

Supplemental data

Unedited blot and gel images

Supplemental table 1

Supplemental video 1

Supplemental video 2

Supporting data values

## Version 1 04/01/2025

Electronic publication

## Version 2 04/22/2025

Hyphen removed from LH2 modified in abstract
