# Supplementary material for: Lysyl hydroxylase 2 glucosylates collagen VI to drive lung cancer progression
Source: J Clin Invest. 2025 Apr 1;135(7):e189197. doi: 10.1172/JCI189197 (PMC11957695; doi:10.1172/JCI189197)
Supplement: Supplemental data [file jci-135-189197-s007.pdf]

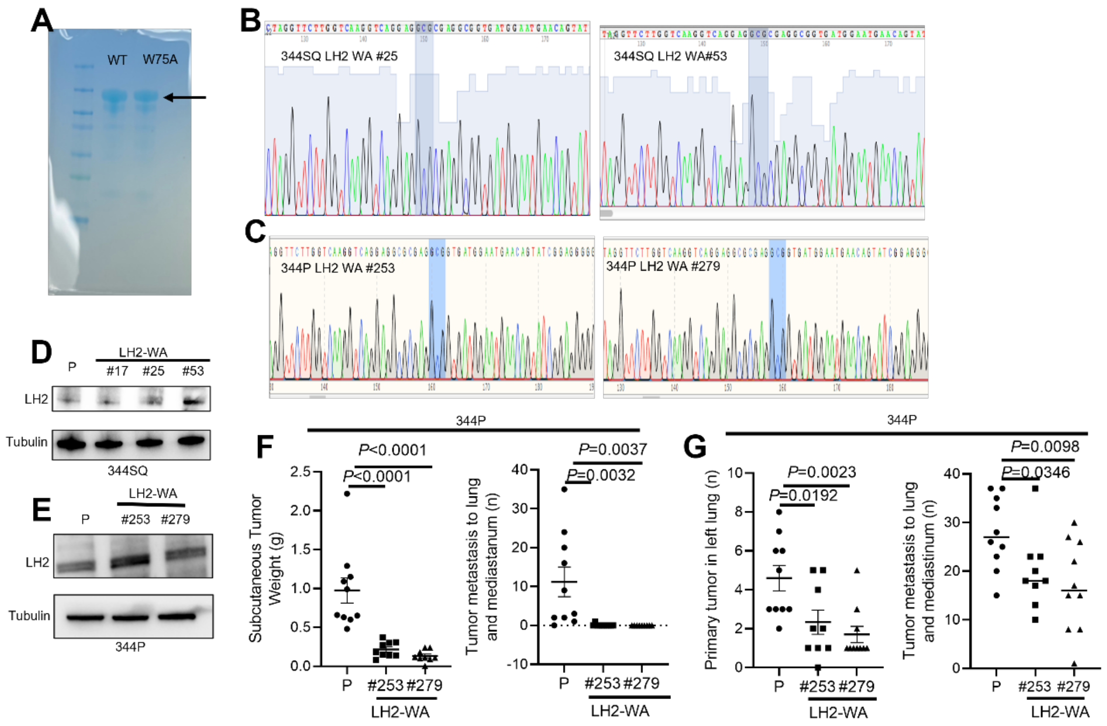

Supplemental Figure 1. The GGT domain of LH2 drives LUAD progression. **A**. Coomassie blue-stained gel of wild-type (WT) or W75A-mutant LH2 protein purified from 293T cells. **B, C**. Cropped PCR sequencing results for 344SQ cells (B) and 344P cells (C) subjected to Crispr-cas9 editing of Plod2, which encodes LH2. The W75A mutation (GCG) is highlighted. **D, E**. Western blot (WB) confirmation that the W75A mutation does not alter LH2 levels in 344SQ cells (D) and 344P cells (E). **F**. Flank tumor weights (left dot plot) and numbers of metastases (right dot plot) in syngeneic, immunocompetent mice injected with parental (P) or LH2-WA 344P cells. **G**. Orthotopic lung tumors (left dot plot) and metastases to mediastinal nodes and contralateral lung (right dot plot) in syngeneic, immunocompetent mice injected with parental (P) or LH2-WA 344P cells. *P* values were determined using one-way ANOVA.

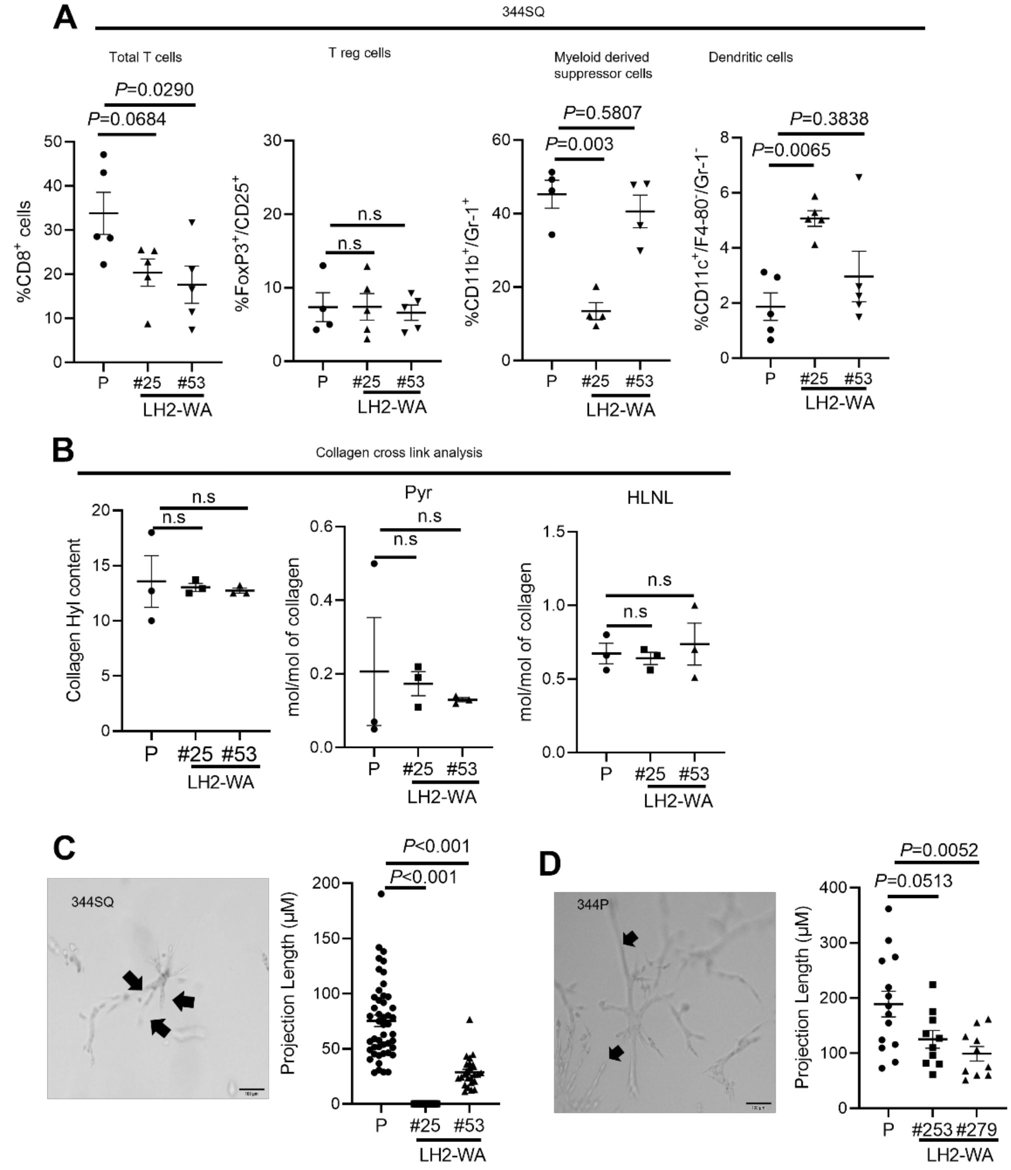

Supplemental Figure 2. The GGT domain of LH2 influences intra-tumoral collagen cross-linking but not the immune microenvironment. **A**. Flow cytometric quantification of immune cell subsets in subcutaneous tumors (dots) generated by parental (P) or W75A-mutant 344SQ cells in syngeneic, immunocompetent mice. **B**. Hydroxylysine (Hyl), pyridinoline (Pyr), and hydroxylysinonorleucine (HLNL) content in collagen samples isolated from flank tumors (dots). Hyl content calculated per 300 hydroxyproline (Hyp) residues per collagen molecule. **C, D**. Invasion assays on 344SQ cells (C) and 344P cells (D) growing in 5% Matrigel mixed with 1 mg/mL type I collagen. Lengths of invasive projections (arrows) per cell (dot) were quantified. Parental (P). LH2-WA. n.s. (not significant). *P* values were determined using one-way ANOVA.

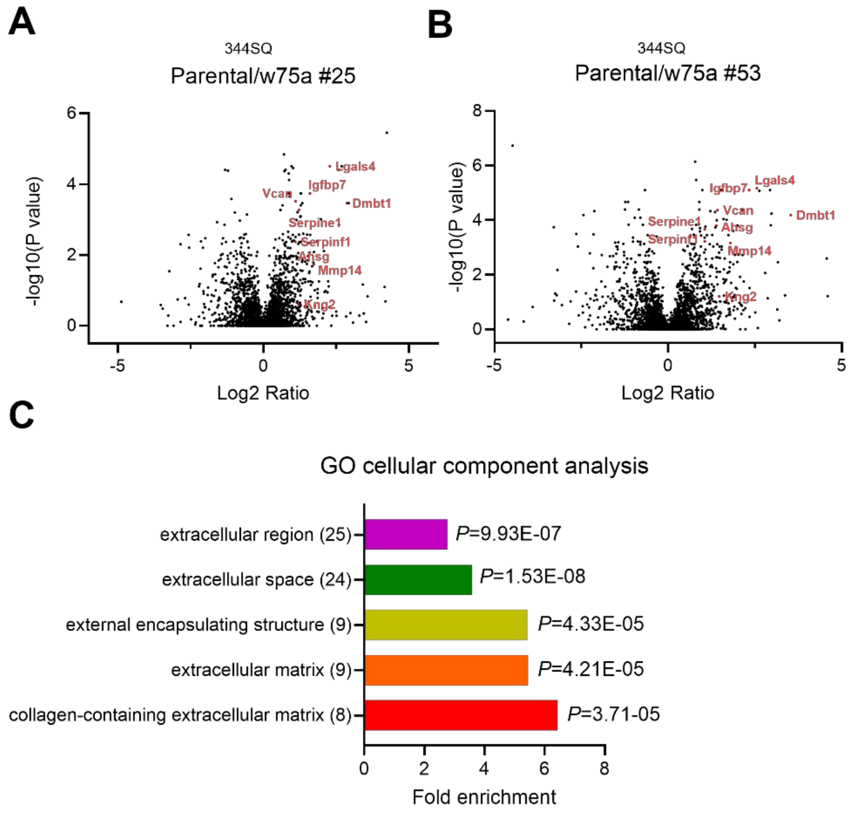

Supplemental Figure 3. The GGT domain of LH2 influences the intra-tumoral extracellular matrix. **A, B**. Volcano plot of proteins (dots) identified by LC-MS analysis of flank tumors generated by parental, W75A clone #25 (A), or W75A clone #53 (B) 344SQ cells. Results are expressed as a $\log_2$ ratio (parental/WA-mutant). y axis: *P* values; x axis: fold change (FC). Locations of a subset of differentially expressed proteins are indicated. **C**. Gene Ontology (GO) term enrichment analysis of proteins downregulated in GGT-inactive tumors (n=51).

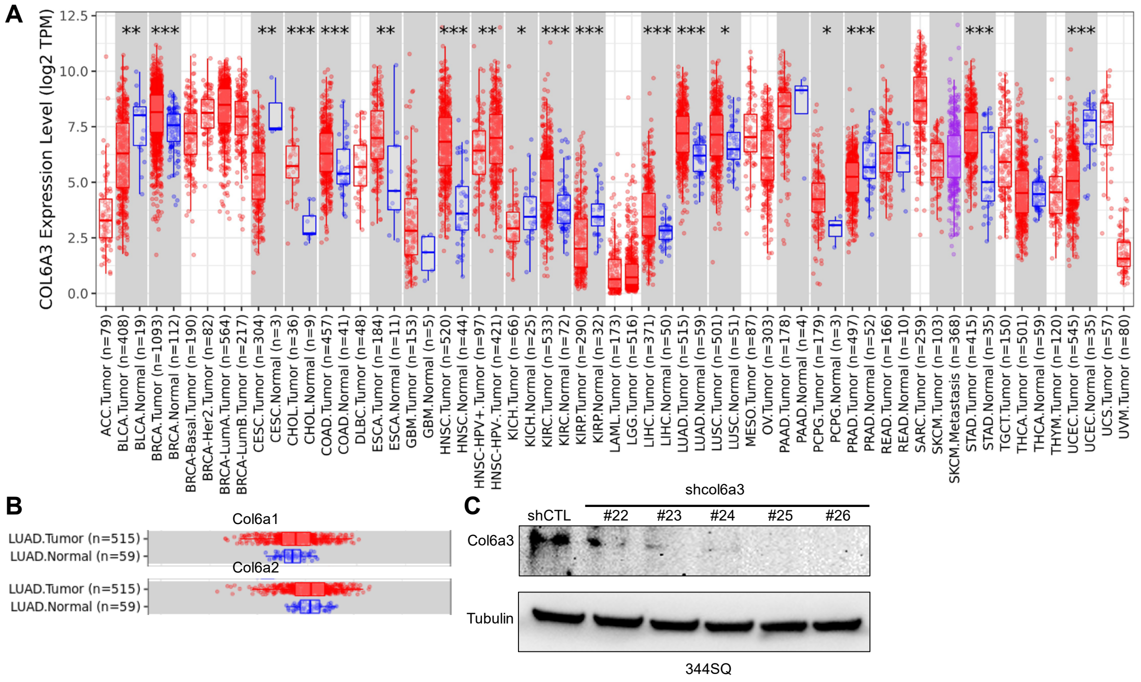

Supplemental Figure 4. Analysis of Col6a3 levels in TCGA pan-cancer cohort. **A**. Col6a3 mRNA levels in malignant and matched normal tissues. Levels are higher in most tumor types, including LUAD. TCGA data analyzed by TIMER2.0 (http://timer.cistrome.org/). **B**. Analysis of Col6a1 and Col6a2 levels in TCGA LUAD cohort. TCGA data analyzed by TIMER2.0 (http://timer.cistrome.org/). **C**. WB confirmation of target gene depletion in Col6a3 shRNA-transfected 344SQ cells.

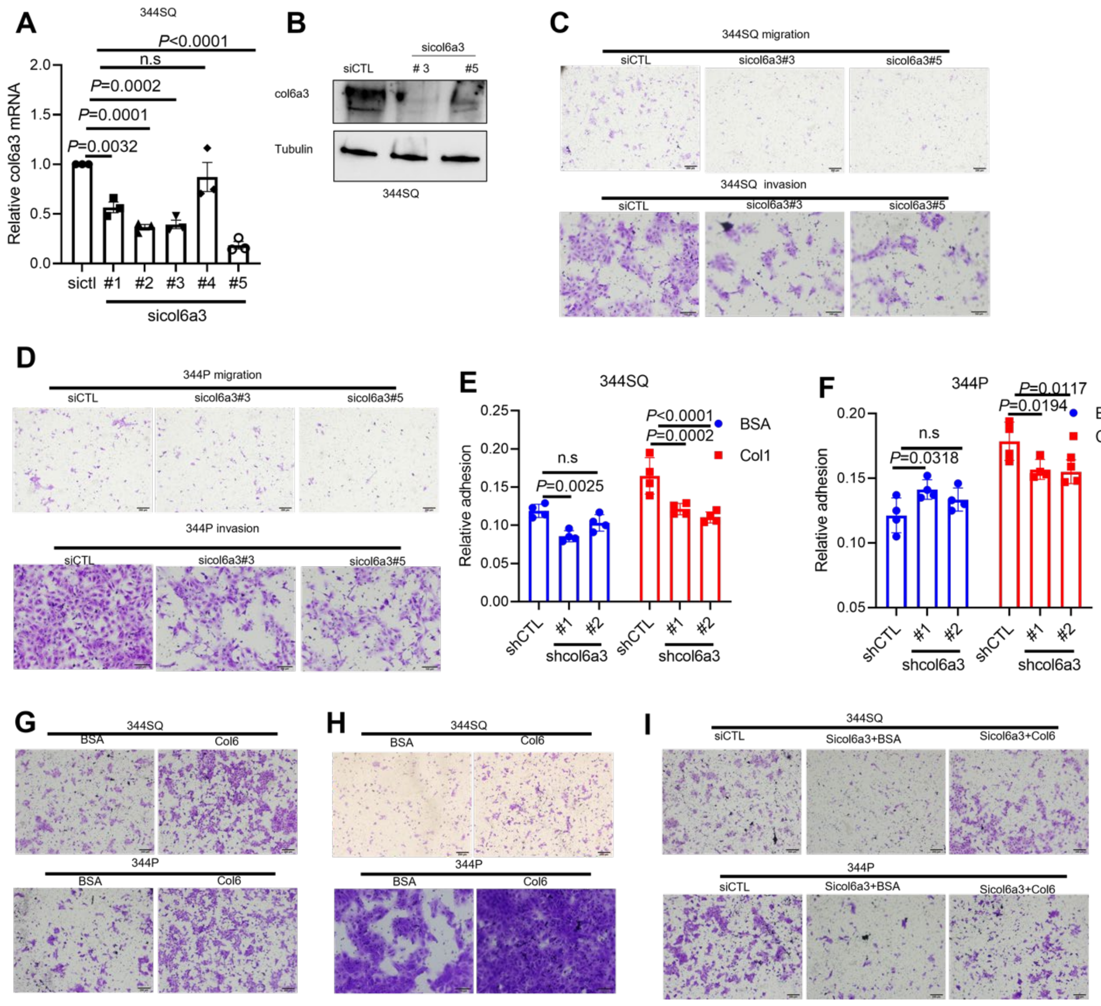

Supplemental Figure 5. Col6a3 depletion inhibits LUAD cell metastatic properties. **A**, **B**. Quantitative PCR (**A**) and WB (**B**) confirmation of target gene depletion in Col6a3 siRNA-transfected 344SQ cells. **C**, **D**. Representative images of migrated cells (top) and invaded cells (bottom) using 344SQ cells (C) and 344P cells (D) in Boyden chambers. **E**,**F** Adhesion assays on siRNA-transfected 344SQ cells (E) and 344P cells (F) seeded on BSA- or type I collagen-coated surfaces. **G**. Representative images of migrated 344SQ cells (top) and 344P cells (bottom) treated with soluble Col6 or BSA. **H**. Representative images of migrated 344SQ cells (top) and 344P cells (bottom) seeded on Col6- or BSA-coated surface. **I**. Representative images of migrated siRNA-transfected 344SQ cells (top) and 344P cells (bottom) treated with soluble Col6 or BSA. For adhesion assays, mean values were calculated from replicate wells (dots). n.s. (not significant). *P* values were determined using, one-way ANOVA (A) or two-way ANOVA (E, F).

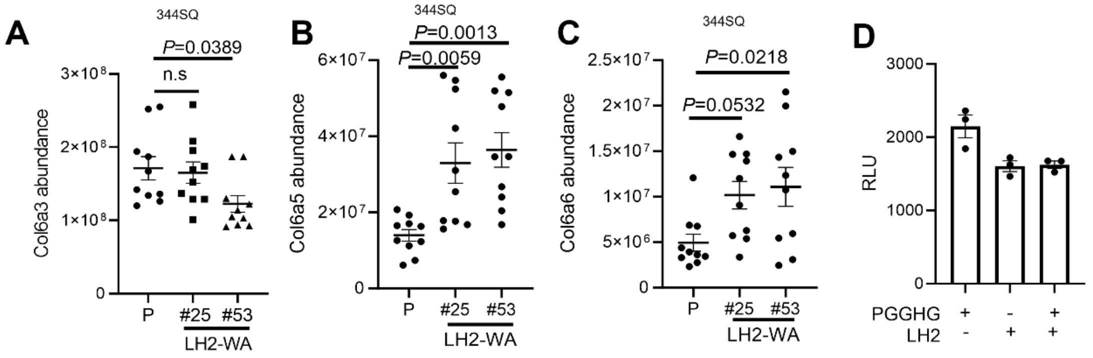

Supplemental Figure 6. GGT inactivation induces upregulation of Col6a5 and Col6a6. **A-C**. Total Col6a3 (A), Col6a5 (B), and Col6a6 (C) protein levels quantified by LC-MS analysis of tumor tissue samples (dots) generated by parental (P) or LH2-WA 344SQ cells. **D.** In vitro GGT activity assay on LH2 reacted with PGGHG to exclude PGGHG as a LH2 substrate. n.s. (not significant). *P* value was analyzed using one-way ANOVA.

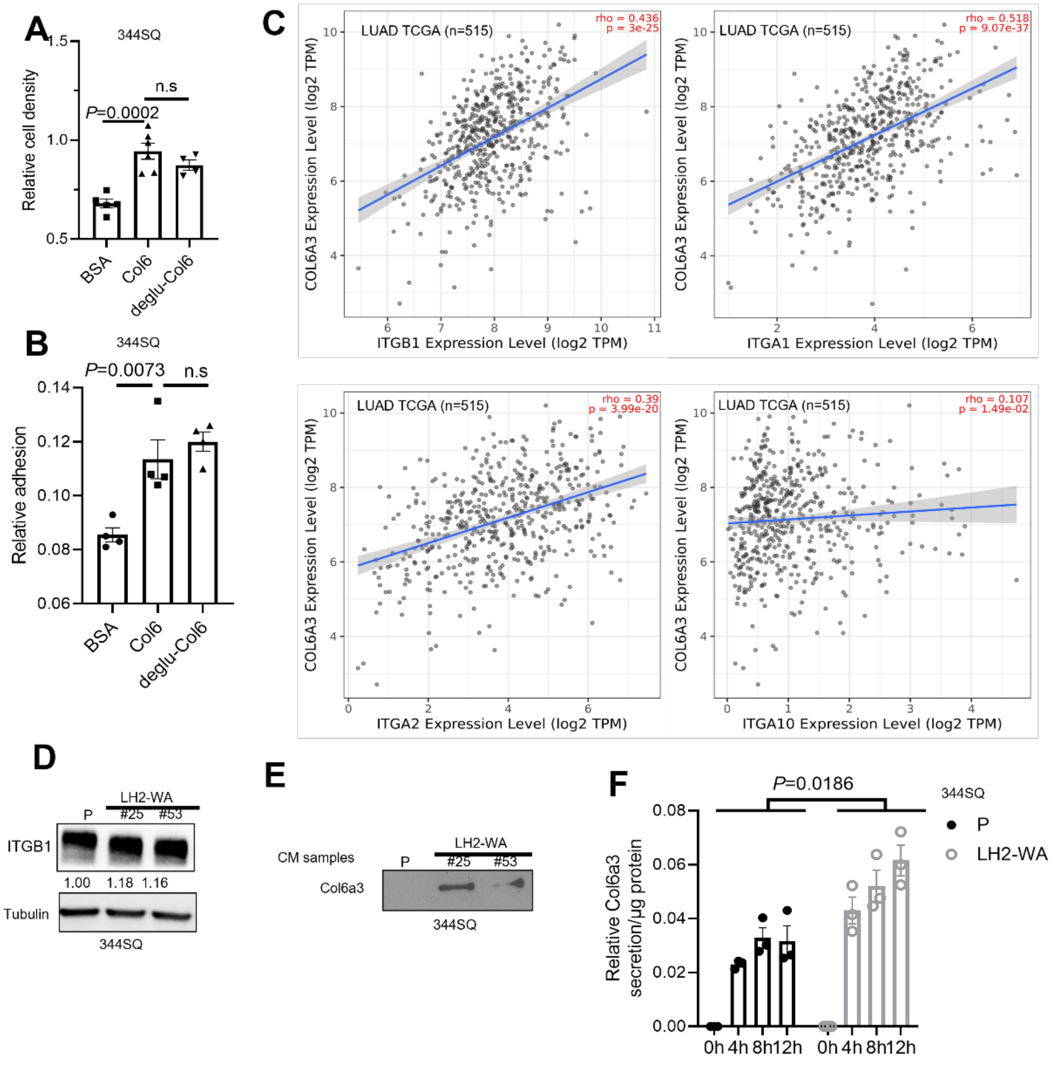

Supplemental Figure 7. Relationships between LH2, Col6, and Col6 receptors in LUAD. **A**, **B**. WST-1 proliferation assays (**A**) and adhesion assays (**B**) on 344P cells seeded on surfaces coated with PGGHG-treated Col6 (deglu), untreated Col6 (Col6), or BSA. **C**. Correlation between Col6a3 and ITG family member mRNA levels in TCGA LUAD cohort. Data analysis by TIMER2.0. **D**. WB evidence that ITGβ1 levels are not altered by LH2-WA mutations in 344SQ cells. **E**. WB analysis of Col6a3 levels in conditioned medium (CM) samples from parental (P) and LH2-WA 344SQ cells **F**. Elisa analysis of Col6a3 levels in conditioned medium (CM) samples from parental (P) and LH2-WA 344SQ cells at different time point. n.s. (not significant). *P* value was analyzed using one-way ANOVA (A, B) and two-way ANOVA (F).

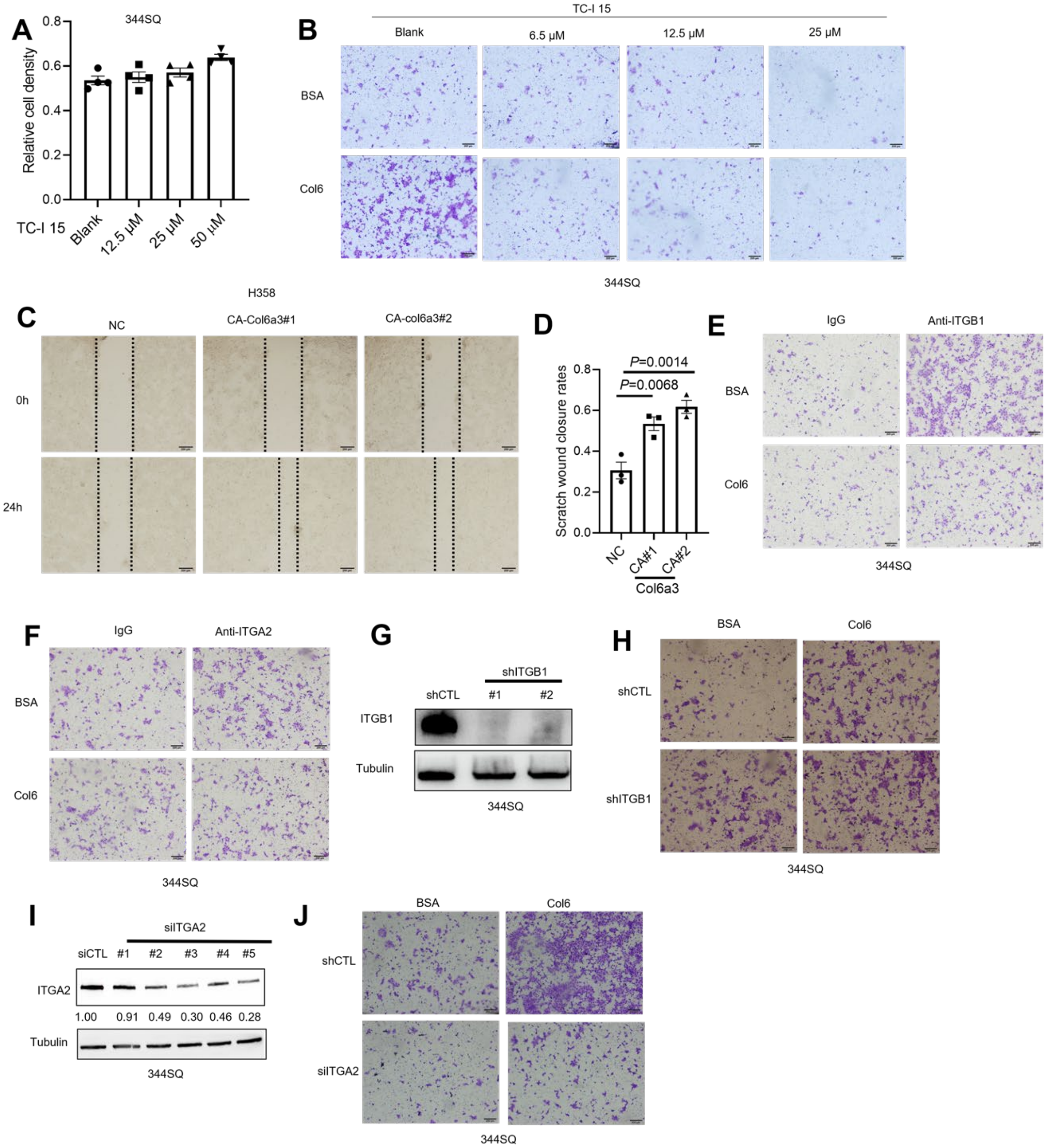

Supplemental Figure 8. Col6a3 drives LUAD cell migration through ITGα2/β1. **A**. WST-1 cell proliferation assay on 344SQ cells treated with ITGα2 inhibitor TC-I 15. **B**. Representative images of migrated 344SQ cells treated with ITGα2 inhibitor TC-I 15 in the presence of soluble Col6 or BSA. **C**. Representative images of scratch wounds in CA-Col6a3 H358 cells. **D**. Scratch wound closure rates calculated for C. **E, F** Representative images of migrated 344SQ cells treated with neutralizing antibodies against ITGβ1 (E) or

ITGα2 (F) followed by soluble Col6 or BSA.**G**. WB confirmation of target gene depletion in 344SQ cells transfected with shRNAs against ITG-β1. **H**. Representative images of shITGβ1 transfected 344SQ cells treated with soluble Col6 or BSA. **I**. WB confirmation of target gene depletion in 344SQ cells transfected with siRNAs against ITGα2. Densitometric values indicated under gels. **J**. Representative images of siITGα2 transfected 344SQ cells treated with soluble Col6 or BSA. *P* values were determined using one-way ANOVA.

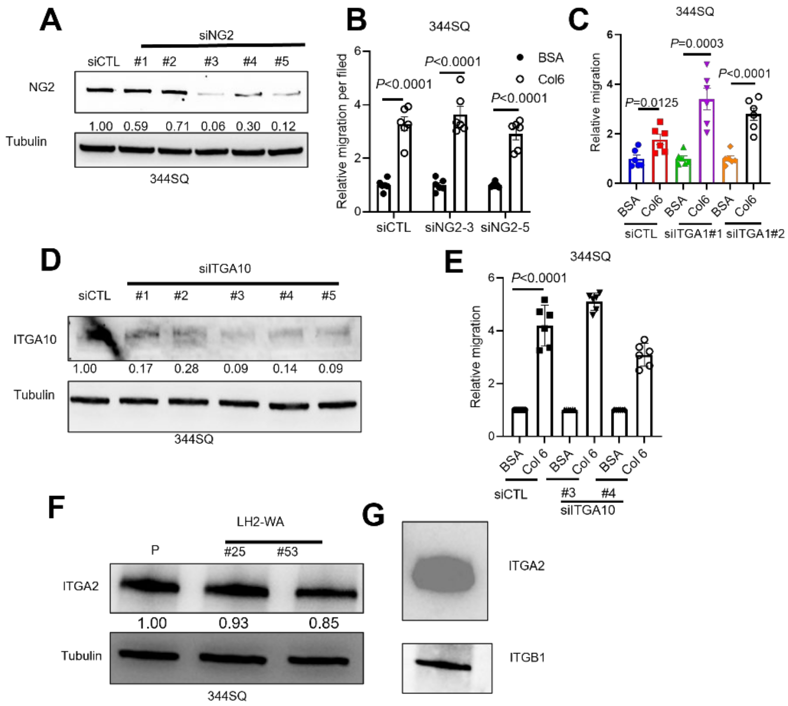

Supplemental Figure 9. Col6 does not drive cell migration through NG2, ITG-α1, or ITG-α10. **A.** WB confirmation of target gene depletion in 344SQ cells transfected with siRNAs against NG2. Densitometric values indicated under gels. **B, C**. Boyden chamber migration assays on siNG2- (B) or siITGα1- (C) 344SQ cells treated with soluble Col6 or BSA. **D**. WB confirmation of target gene depletion in siITGα10-transfected 344SQ cells. Densitometric values indicated under gel. **E**. Boyden chamber migration assays on siITGα10-transfected 344SQ cells treated with soluble Col6 or BSA. **F.** WB analysis of ITGα2 in parental (P) and LH2-WA 344SQ cells. Densitometric values indicated under gels. **G**. WB evidence that ITGα2 purified from 293FT cells is in a heterodimeric complex with ITGβ1. *P* values were determined using Student two-tailed t-test.

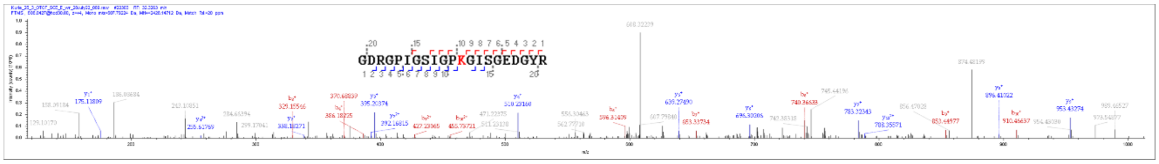

Supplemental Figure 10. Glycosylated Col6a3 peptide ([R].GDRGPIGSIGPKGISGEDGYR.[G]) detected by LC/MS of flank tumor samples.

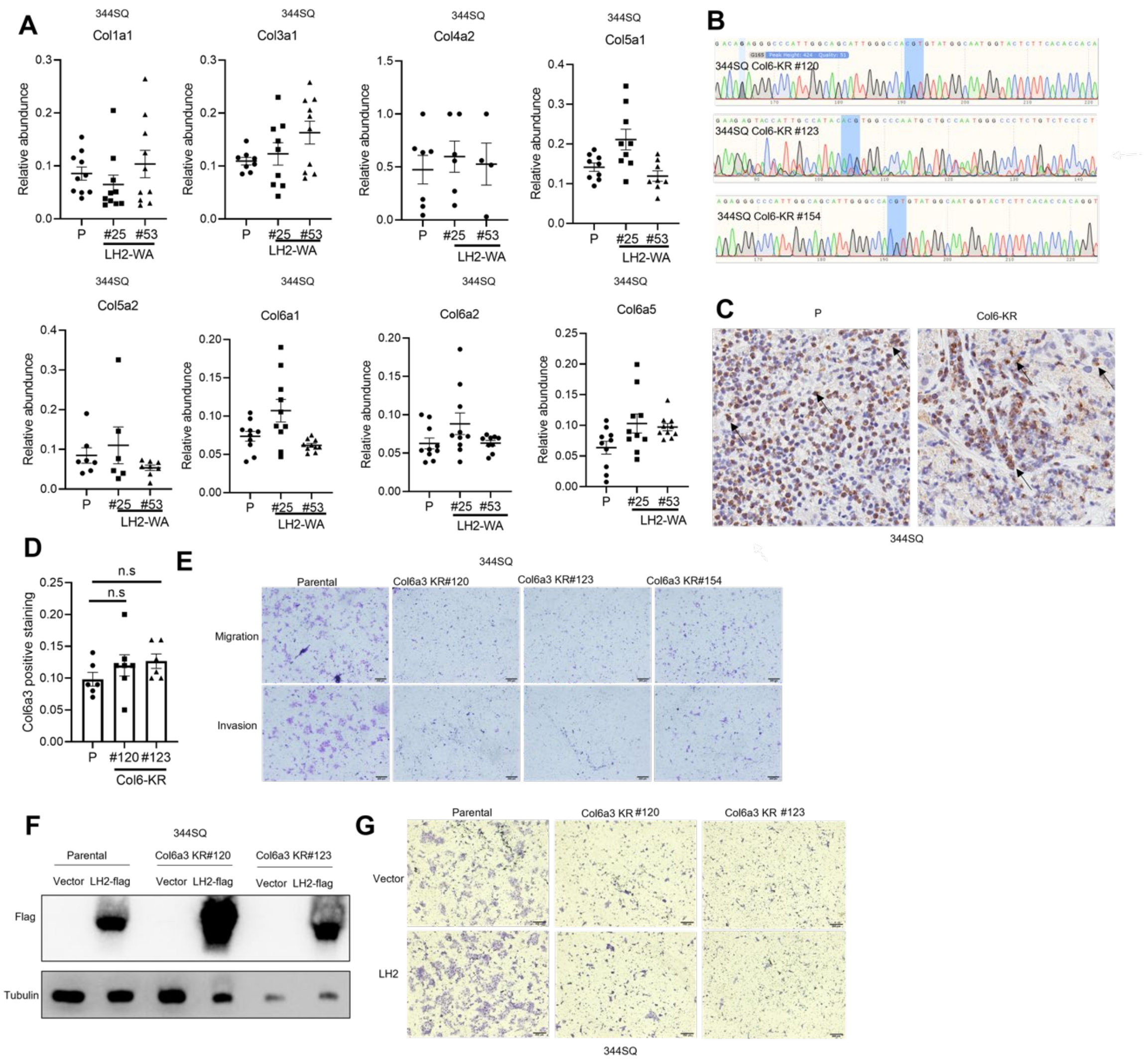

Supplemental Figure 11. Relationships between LH2 GGT activity, Col6a3 glucosylation, and total collagen levels. **A**. GG-Hyl levels on collagen family members in parental (P) and LH2-WA 344SQ tumor samples (dots). **B**. Cropped PCR sequencing results for 344SQ cells subjected to Crispr-cas9 editing of Col6a3. The K2049R mutation (CGT) is highlighted. **C, D**. Immunohistochemical staining (C) of tumors generated by parental or Col-KR 344SQ cells. Col6a3-positive cells in (C) quantified by image analysis (ImageScope, Positive Pixel Count V9) (D). Original magnification, ×10. **E**. Representative images of migrated 344SQ cells (top) and invasive 344SQ cells (bottom). **F**. WB analysis to detect flag-tagged LH2 in parental (P) or Col6a3-KR 344SQ cells. **G**. Representative images of migrated cells in Boyden chambers. Parental (P) and Col6-KR 344SQ cells

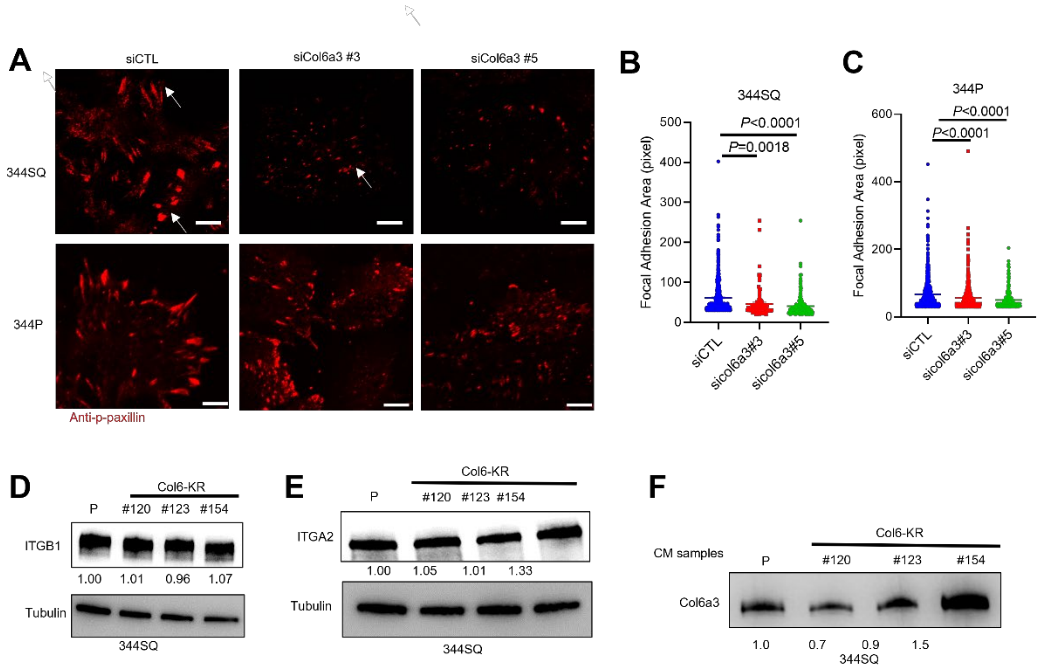

Supplemental Figure12. Col6-KR mutations do not decrease Col6 secretion or ITG expression levels. **A**. FAs (arrows) in siRNA-transfected 344SQ cells and 344P cells were detected by anti-p-paxillin antibody staining. Scale bar: 10 µm. **B**, **C**. Areas of FAs (dots) determined for 344SQ cells (B) and 344P cells (C) (n≥50 per group). **D-F**. WB evidence that Col6-KR mutations do not influence ITGβ1 levels (D), ITGα2 levels (E), or Col6a3 secretion in conditioned medium (CM) samples (F). *P* values were determined using one-way ANOVA test.

Supplemental table 2. Efficiency of deglycosylation of glycosylated Col6a3 peptides by PGGHG.

GG-Hyl peptides of Col6a3	Efficiency of deglucosylation by PGGHG
[R].RGNSGPPGIVGQKGDPGYPGPAGPK.[G]	47.8 %
[R].GNSGPPGIVGQKGDPGYPGPAGPKGNR.[G]	11.6%
[R].GPKGETGDLGPMGVPGRDGVPGGPGETGK.[N]	23%
[R].GPPGAKGNKGGPGQPGFEGEQGTR.[G]	48.6%
[R].GDPGNPGQDSQERGPKGETGDLGPMGVPGR.[D]	67.1%
[R].KGEPGEPGPKGGIGNR.[G]	32.1%
[R].RGNSGPPGIVGQKGDPGYPGPAGPK.[G]	32.7%
[R].GFPGEKGEVGEIGLDGLDGEDGDKGLPGSSGEK.[G]	36.%
[K].GEPGEPGPKGGIGNR.[G]	27.3%
[R].GPIGSIGPKGIPGEDGYR.[G]	73.7%
[K].GLPGSSGEKGNPGR.[R]	78.1%
[R].GPKGETGDLGPMGVPGR.[D]	75.5%
[R].GDRGPIGSIGPKGIPGEDGYR.[G]	43.9%

Supplemental table 3. List of glycosylated peptide of collagen in tumor tissues detected by LC-MS.

Collagen type	GG-Hyl peptides
Col12a1	[R].RNNVILQPLQPDTPYKITVIAIYEDGDGGHLTGNGR.[T]
Col6a3	[R].GDRGPIGSIGPKGISGEDGYR.[G]
Col6a3	[R].GPIGSIGPKGISGEDGYRGYPGDEGGPGER.[G]
Col6a3	[R].GFPGEKGELGEIGLDGLDGEEGDK.[G]
Col6a3	[R].GPKGETGDIGPMGLPGR.[D]
Col6a5	[K].DLGICVLALGIGDVYKEQLLPITGNSEKIITFR.[D]
Col6a5	[R].GQKGVKGFSGAQGEHGEDGLDGLDGEEGFYGFR.[G]
Col6a5	[R].RGPKGTAGQPIYSPCELIQFLR.[D]
Col6a5	[R].GPKGTAGQPIYSPCELIQFLR.[D]
Col1a1	[R].GLPGTAGLPGMKGHR.[G]
Col1a1	[R].GEAGPPGPAGFAGPPGADGQPGAKGEPGDTGVKGDAGPPGPAGPAGPPGPIGNVGAPGPK.[G]
Col14a1	[R].GPKGQQGEQGPKGPEGPR.[G]
Col6a1	[R].EGPVGIPGDSGEAGPIGPKGYR.[G]
Col6a6	[K].KGPPGFKGSDGYLGEEGIAGER.[G]
Col6a2	[K].VSCLEIPGPHGPKGYR.[G]
Col6a2	[R].GLAGEVGSKGAKGDR.[G]
Col6a2	[R].GDFGLKGTPGR.[K]
Col7a1	[R].GEKGEAALTEDDIRDFVR.[Q]
Col3a1	[K].GPAGMPGFPGMKGHR.[G]
Col3a1	[R].GSPGPQGIKGESGKPGASGHNGERGPPGPQGLPGQPGTAGEPGR.[D]
Col5a1	[R].GFDGLAGLPGEKGHR.[G]

Col5a1	[R].GGPNGDPGPLGPTGEKGK.[L]
Col5a1	[R].GQQGLFGQKGDEGSR.[G]
Col5a2	[R].KGQKGEPGLVPVVTGIR.[G]
Col4a2	[R].GEKGTPGVAGVFGETGPTGDFGDIGDTVDLPGSPGLKGER.[G]

Supplemental table 4. List of gRNA and ssODN utilized in the CRISPR/CAS9 gene editing.

mPLOD2 W75A gRNA 1	GGTCAAGGTCAGGAGTGGAG
mPLOD2 W75A gRNA 2	TTCTTGGTCAAGGTCAGGAG
mcol6a3 K2049R gRNA	GAGUACCAUUGCCAUACCUU
mPLOD2 W75A ssODN	CAGTAATCTCACCTTCTGGCCCCCTCCGATACTGTTCATTCCATCACCGCCT CGCGCCTCCTGACCTTGACCAAGAACCTAGAAATGAAATCAAGAGCTCATC CTGAGG
mcol6a3 K2049R ssODN	CAAATAGTGTCAGAGCTGCATGTCTAACCTGTGGTGTGAAGAGTACCATTG CCATACACGTGGCCCAATGCTGCCAATGGGCCCTCTGTCTCCCCTCTCTCC AGAGCACTTG

Supplemental table 5. List of the primers utilized in the study.

mPlod2-W75-PCR-forward	ACCCTCCCGTGCGTTAATTT
mPlod2-W75-PCR-reverse	TGCACACTCAAGCATGACCT
mcol6a3 K2049R PCR	ACCTTGGAGCCCAGAATTGG
hPLOD2 PCR forward	TCCCCCGGGGCCACCATGGGGGATGCACGGTGAA
hPLOD2 PCR reverse	CTAGTCTAGATTACTTGTCGTCATCGTCTTTGTAGTCGGGATCTATAAA TGACACTGC
hPLOD2 W75A PCR forward	GGAGAAGAAGCGAGAGGTGGTG
hPLOD2 W75A reverse	CACCACCTCTCGCTTCTTCTCCTTGA
mcol6a3 QPCR forward	CCACAGCCAGACCTGCATTA
mcol6a3 QPCR reverse	GGAGTCTGGCACTGTTCTCC
mL32 QPCR forward	AGAGGACCAAGAAGTTCATCAG
mL32 QPCR reverse	CCAGCTCCTTGACATTGTGG
