# Supplementary material for: Lysyl hydroxylase 2 glucosylates collagen VI to drive lung cancer progression
Source: J Clin Invest. 2025 Apr 1;135(7):e189197. doi: 10.1172/JCI189197 (PMC11957695; doi:10.1172/JCI189197)

Full unedited blot/gel for Figure 2C

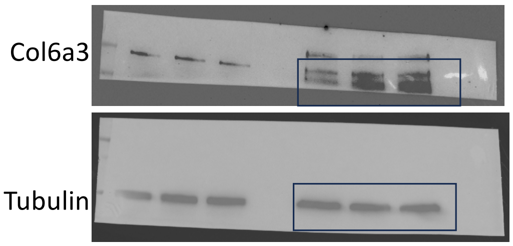

Full unedited blot/gel for Figure 4A

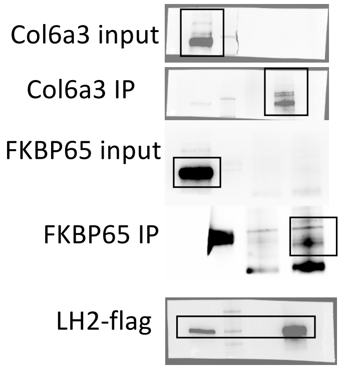

Full unedited blot/gel for Figure 4G

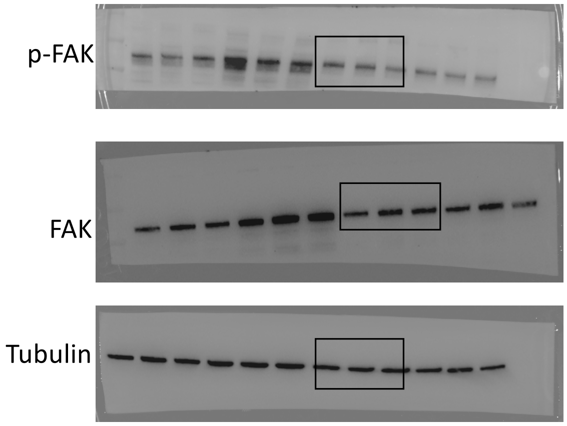

Full unedited blot/gel for Figure 6G

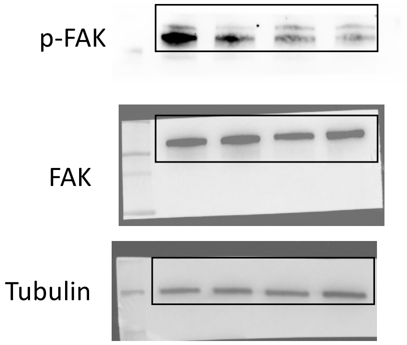

Full unedited blot/gel for Figure 6H

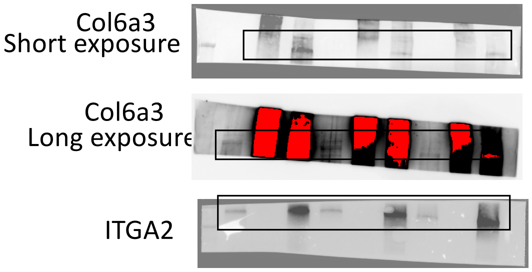

Full unedited blot/gel for Fig. S1D

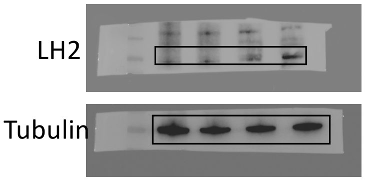

Full unedited blot/gel for Fig. S1E

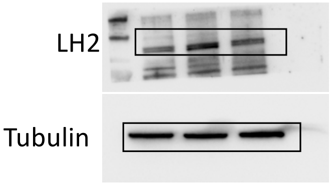

Full unedited blot/gel for Fig. S4C

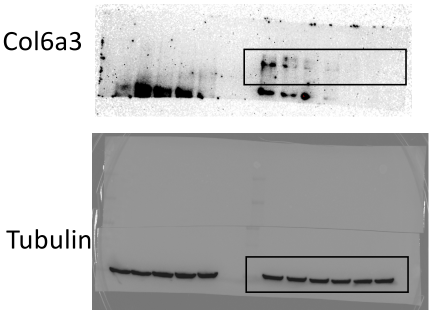

Full unedited blot/gel for Fig. S5B

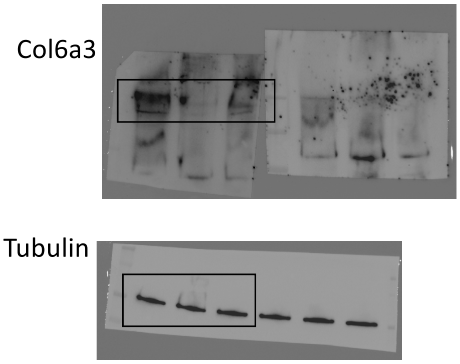

Full unedited blot/gel for Fig. S7D

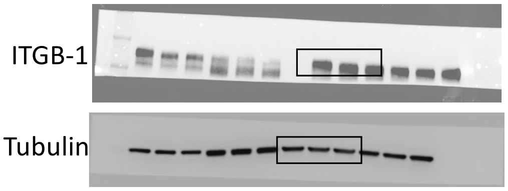

Full unedited blot/gel for Fig. S7E

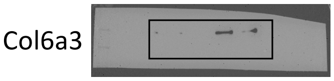

Full unedited blot/gel for Fig. S8G

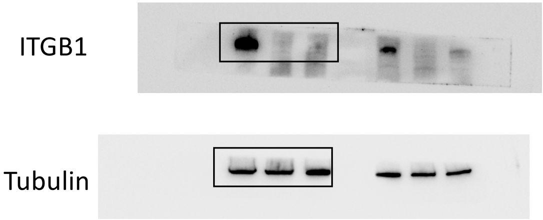

Full unedited blot/gel for Fig. S8I

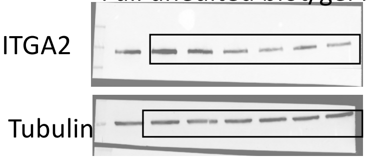

Full unedited blot/gel for Fig. S9A

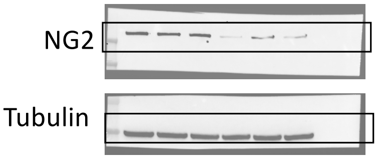

Full unedited blot/gel for Fig. S9D

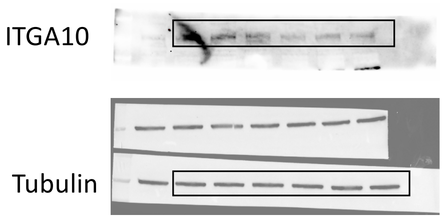

Full unedited blot/gel for Fig. S9F

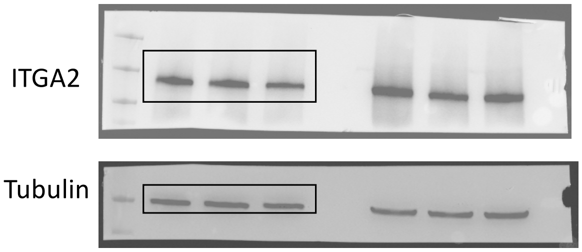

Full unedited blot/gel for Fig. S9G

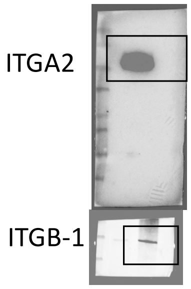

Full unedited blot/gel for Fig. S11F

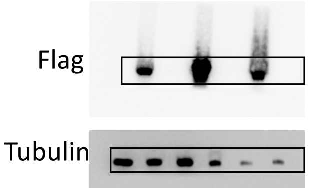

Full unedited blot/gel for Fig. S12D and E

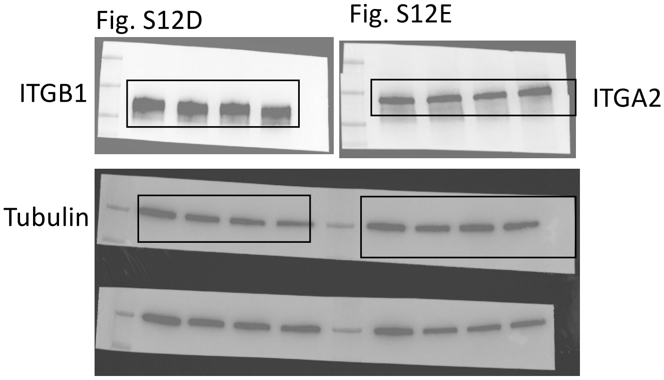

Full unedited blot/gel for Fig. S12F

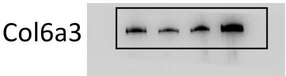

Supplement: Unedited blot and gel images [file jci-135-189197-s008.pdf]
